# Diagnostic Imaging in Intervertebral Disc Disease

**DOI:** 10.3389/fvets.2020.588338

**Published:** 2020-10-22

**Authors:** Ronaldo C. da Costa, Steven De Decker, Melissa J. Lewis, Holger Volk, The Canine Spinal Cord Injury Consortium (CANSORT-SCI), Sarah A. Moore

**Affiliations:** Author Affiliations: Department of Veterinary Clinical Sciences, The Ohio State University College of Veterinary Medicine, Columbus, OH, United States; Department of Clinical Sciences, North Carolina State University College of Veterinary Medicine, Raleigh, NC, United States; Department of Small Animal Clinical Sciences, College of Veterinary Medicine and Biomedical Sciences, Texas A&M University, College Station, TX, United States; Department of Veterinary Clinical Sciences, Purdue University College of Veterinary Medicine, West Lafayette, IN, United States; Professor in Small Animal Clinical Sciences, College of veterinary Medicine, Texas A&M University, TX, United States; Department of Veterinary Clinical Sciences, College of Veterinary Medicine, The Ohio State University, Columbus, OH, United States; Department of Clinical Sciences, Colorado State University, Fort Collins, CO, United States; Department of Clinical Science and Services, Royal Veterinary College, Hertfordshire, United Kingdom; The Royal Veterinary College, University of London, Hertfordshire, United Kingdom; CVS referrals, Bristol Veterinary Specialists at Highcroft, Bristol, United Kingdom; Faculty of Veterinary Medicine, Institute of Veterinary Pathology, Leipzig University, Leipzig, Germany; Division of Clinical Neurology, Department for Clinical Veterinary Medicine, Vetsuisse Faculty, University of Bern, Bern, Switzerland; Department of Small Animal Medicine and Surgery, University of Veterinary Medicine Hannover, Hannover, Germany/Europe; Department of Veterinary Medicine and Surgery, University of Missouri, Columbia, MO, United States; Department of Small Animal Medicine and Surgery, University of Veterinary Medicine Hannover, Hannover, Germany/Europe.; ^1^Department of Veterinary Clinical Sciences, The Ohio State University, Columbus, OH, United States; ^2^Department of Clinical Sciences and Services, Royal Veterinary College, London, United Kingdom; ^3^Department of Veterinary Clinical Sciences, Purdue University College of Veterinary Medicine, West Lafayette, IN, United States; ^4^Department of Small Animal Medicine and Surgery, University of Veterinary Medicine Hanover, Hanover, Germany

**Keywords:** herniation, extrusion, protrusion, computed tomography, magnetic resonance imaging, intervertebral disc

## Abstract

Imaging is integral in the diagnosis of canine intervertebral disc disease (IVDD) and in differentiating subtypes of intervertebral disc herniation (IVDH). These include intervertebral disc extrusion (IVDE), intervertebral disc protrusion (IVDP) and more recently recognized forms such as acute non-compressive nucleus pulposus extrusion (ANNPE), hydrated nucleus pulposus extrusion (HNPE), and intradural/intramedullary intervertebral disc extrusion (IIVDE). Many imaging techniques have been described in dogs with roles for survey radiographs, myelography, computed tomography (CT), and magnetic resonance imaging (MRI). Given how common IVDH is in dogs, a thorough understanding of the indications and limitations for each imaging modality to aid in diagnosis, treatment planning and prognosis is essential to successful case management. While radiographs can provide useful information, especially for identifying intervertebral disc degeneration or calcification, there are notable limitations. Myelography addresses some of the constraints of survey radiographs but has largely been supplanted by cross-sectional imaging. Computed tomography with or without myelography and MRI is currently utilized most widely and have become the focus of most contemporary studies on this subject. Novel advanced imaging applications are being explored in dogs but are not yet routinely performed in clinical patients. The following review will provide a comprehensive overview on common imaging modalities reported to aid in the diagnosis of IVDH including IVDE, IVDP, ANNPE, HNPE, and IIVDE. The review focuses primarily on canine IVDH due to its frequency and vast literature as opposed to feline IVDH.

## Introduction

Intervertebral disc disease (IVDD) is the most common spinal cord disease of dogs, being responsible for 2.3–3.7% of admissions to veterinary hospitals (1, 2). The diagnosis of all IVDD forms is based on imaging, with the imaging techniques evolving over the years.

Even though IVDD was first reported in a paraplegic Dachshund by Dexler in 1896, it was truly well-characterized by Olsson and Hansen in the early 1950s (3). In 1952, Hansen classified IVDD into acute, or Hansen type I intervertebral disc extrusion (IVDE), and chronic, or Hansen type II intervertebral disc protrusion (IVDP) (4). Over the years and primarily with the widespread use of magnetic resonance imaging (MRI), newer forms began to be recognized, such as acute non-compressive nucleus pulposus extrusion (ANNPE), hydrated nucleus pulposus extrusion (HNPE) and intradural/intramedullary intervertebral disc extrusion (IIVDE) (5, 6). In addition, intervertebral disc (IVD) degeneration and protrusion are also associated with other important spinal conditions such as cervical spondylomyelopathy and degenerative lumbosacral stenosis (7, 8). A study with 677,000 dogs suggested that the overall prevalence of all intervertebral disc degeneration-related diseases was 27.8% (1).

The most commonly described clinical form of intervertebral disc herniation (IVDH) is IVDE, which leads to acute spinal pain and variable degrees of paresis up to paralysis. Radiography was the most widely used method for the diagnosis of IVDD starting in the 1950s and extending until the 1980s (4, 9–11).

Myelography started to be used in the 1960s and 1970s (12, 13), but publications utilizing this technique in dogs remained variable until the early to mid 1990s (14–16). Starting in the 1990s, published case series began to have all dogs confirmed with myelography (17), which then became the norm in the publications from the 2000s onward (18). It was also in the 2000s that magnetic resonance imaging (MRI) and computed tomography (CT) started to become routinely used in referral hospitals. Since that time, most publications have focused on descriptive and comparative studies involving multiple modalities, typically either CT or MRI, or both.

A 2016 study looked at the practice patterns of diplomates of the American College of Veterinary Internal Medicine in the specialty of Neurology and American College of Veterinary Surgeons for the diagnosis of IVDH in North America. Among the board-certified neurologists, MRI was the most commonly used technique (75%), whereas among the board-certified surgeons, CT with or without concurrent myelography was the most commonly used imaging modality (58%). Approximately 28% of board-certified surgeons used MRI. Myelography alone was chosen as the most commonly used modality by 14% of board-certified surgeons, and <1% of board-certified neurologists (19). The imaging preferences in other countries or continents are unknown, although, overall, CT scanners are far more widely available than MRI.

As the saying goes “the best treatment is a correct diagnosis,” in order to accurately diagnose all forms of IVDD and institute appropriate treatment, correct identification of affected disc sites, extension and lateralization are crucial for treatment planning. In this manuscript, we will review all imaging modalities used to presumptively or accurately diagnose all forms of IVDD.

## Imaging Diagnosis of Acute Intervertebral Disc Extrusion (IVDE)

Hansen type I intervertebral disc extrusion (IVDE) is characterized by chondroid degeneration of the gelatinous nucleus pulposus with transformation to hyaline cartilage and mineralization. Ultimately this process leads to rupture of the annulus fibrosus and herniation of calcified nucleus pulposus into the vertebral canal and/or intervertebral foramen (4, 20). Disc herniation can be a misleading term because it is not the entire disc that herniates but primarily parts of the nucleus pulposus and/or the ruptured annulus. The extruded disc material will cause myelopathic (proprioceptive ataxia, paresis, plegia) and radiculopathic (pain) clinical signs (21).

## Radiography

Orthogonal radiographic projections, lateral and ventrodorsal, are routinely used in the diagnostic investigation of cases suspected of IVDE. The use of general anesthesia has been recommended in order to obtain diagnostic radiographs (22). However, this is very rarely, if ever, done in clinical practice and truly is an unnecessary recommendation. Patients can be properly positioned for radiographs under sedation. Radiographs are a screening test in the diagnostic approach of spinal cases, therefore general anesthesia should be reserved for techniques that can provide a definitive diagnosis of compressive lesions such as myelography, CT or MRI.

Radiographic changes supportive of IVDE are narrowing of the disc space, narrowing of the articular facets, narrowing and/or increased opacity of the intervertebral foramen, presence of mineralized disc material within the vertebral canal and vacuum phenomenon (22, 23). A popular finding, mineralization (also known as calcification) of the disc space *in situ*, is a controversial one. Intervertebral disc mineralization is supportive of intervertebral disc degeneration but not disc extrusion. Radiographic detection of calcification requires significant IVD mineralization to be present, since many discs documented to have mineralization on histopathology were not apparent radiographically (24). Interestingly, a recent study found that neither CT nor low-field MRI had a higher intra- or interobserver agreement compared to radiographs for the detection of disc calcification (25). Studies have shown that disc calcification at 2 years of age was a significant predictor of disc herniation later in life (26) and also a risk factor for recurrent herniation following surgery (27, 28). However, it has been reported that just as many extrusions occurred at intervertebral discs with radiographic evidence of calcification as those without it (29). Additionally, in a prospective MRI study, none of the 65 calcified discs corresponded with the actual site of disc extrusion (30). It must be kept in mind that discs can show evidence of calcification and decalcification naturally, as demonstrated in longitudinal studies in Dachshunds (31). Therefore, using intervertebral disc calcification as a diagnostic criterion would not be appropriate.

In the thoracolumbar region, survey radiographs typically have a reported sensitivity ranging between 51 and 61% (32–36), with only one study reporting a much higher sensitivity at 94.7% using digital radiographs (37). Importantly, radiographic findings can be *suggestive* of thoracolumbar IVDE but are never diagnostic and can also be fraught with significant interobserver variation (23). Radiographs are even less sensitive in the cervical region. Among radiographic changes, intervertebral disc mineralization and narrowing of the affected disc space had the highest correlation with myelography (38). A study evaluated the diagnostic accuracy of radiographs in the diagnosis of cervical IVDE and IVDP using 4 raters (2 radiologists and 2 surgeons) and found an overall accuracy of only 35% (31.3–40.6%). When a suspected abnormal disc space was detected, a higher agreement was found but remained low, at only 58% (53–67%) (39).

## Myelography

Myelography is a radiographic technique in which spinal radiographs are obtained following the injection of a radiopaque contrast agent into the subarachnoid space. It is widely available because it can be performed in any place with an X-ray machine (40, 41). Myelography has been largely replaced by cross-sectional imaging in countries where CT and MRI are routinely available for veterinary patients.

In order to achieve a diagnostic myelographic study, several technical aspects are important. Non-ionic, iodinated, water-soluble contrast agents such as iohexol, iopamiron, or iotrolan, with concentrations ranging from 180 to 240 mg/ml, ideally at body temperature, should be used (42). Injection sites can be either the cerebellomedullary cistern or lumbar region, although lumbar myelography is superior, especially for investigating the thoracolumbar region (21, 43). Lumbar myelography is also overall, safer than cisternal myelography, though it is technically more challenging and is more frequently associated with leakage of contrast into the epidural space. The site of injection site should be the ventral subarachnoid space at L5-6 (ideally). The L4-5 space should be avoided since it is the center of the lumbosacral intumescence in most dogs. In the cerebellomedulary cistern, the dorsal subarachnoid space should be used. Dose of contrast agents ranges 0.3–0.45 ml/kg body weight and should not exceed a total volume >8 ml independent of the dog's size (44). Larger volumes should only be used if the initial injection was not diagnostic. Following injection, lateral, ventrodorsal, +/- oblique projections should be acquired in all cases (45).

**Figure 1:**
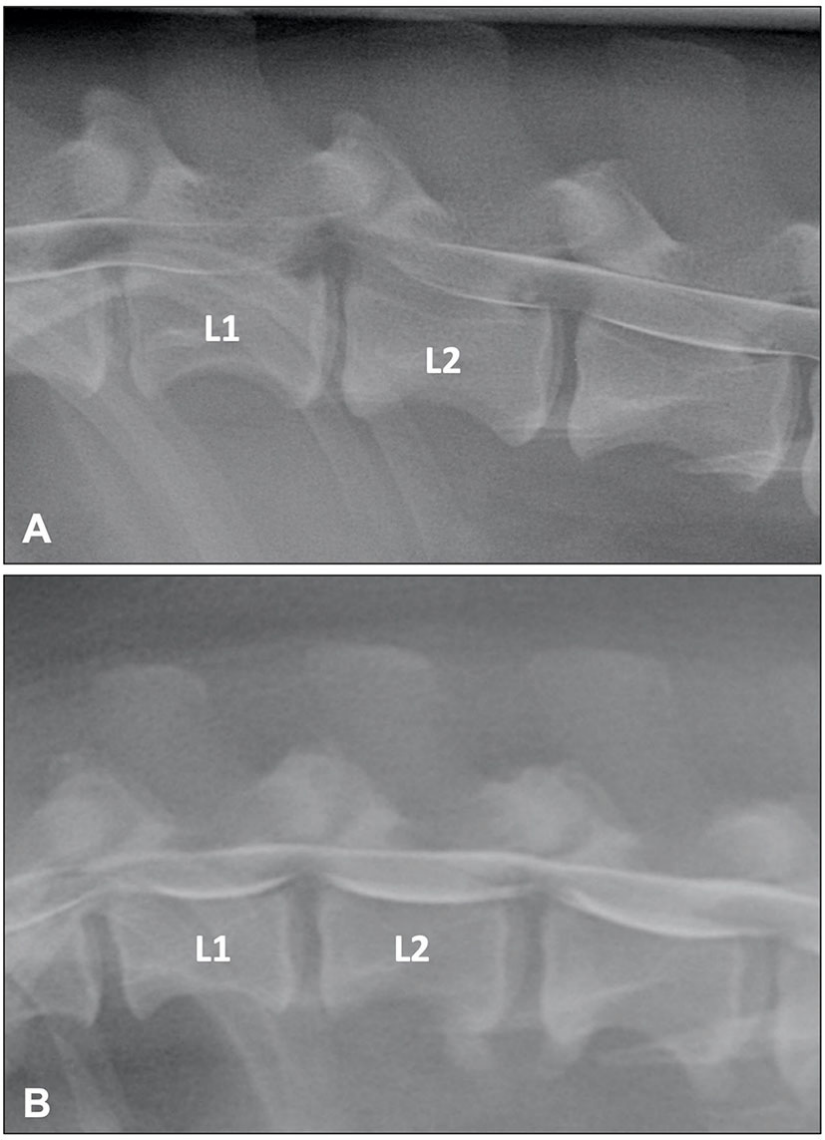
**(A)** Lateral myelogram of a 5-year old Basset Hound with an L1-L2 IVDE. Thinning of the contrast columns is severe and the ventral contrast column is almost discontinued at L1-L2. Thinning of the contrast column is beyond the boundaries of the affected disc and distributed asymmetrical with a longer dorsal deviation of the ventral contrast column caudal to the affected disc. **(B)** Lateral myelogram of a 9-year old Basset Hound with IVDPs between L1-L2 and L2-L3. Thinning of the contrast columns is only mild, dorsal deviation of the ventral contrast column is centered over the affected disc spaces and distribution of contrast thinning is cranio-caudally symmetrical over the disc spaces.

Myelographic patterns of IVDE are those characteristics of an extradural compression. In order to facilitate differentiation from IVDP, the following criteria have been proposed for the diagnosis of IVDE: (1) thinning and deviation of the contrast columns, (2) thinning of the contrast columns is mild to severe or contrast columns are discontinuous, (3) thinning of the contrast columns is diffuse and beyond the boundaries of the affected disc, and (4) asymmetrical distribution of contrast column thinning cranial or caudal to the affected disc (Figure 1) (46). It is important to evaluate the ventrodorsal and/or oblique views to determine the presence of axial deviation of the contrast to determine lateralization of the lesion and guide the surgical approach (21, 45, 47). It has been proposed that extensive intramedullary patterns were associated with a poor outcome (48); however, this was not confirmed by a different study (49). Extensive spinal cord swelling with evidence of contrast medium infiltration into the spinal cord has been reported as an indication of myelomalacia (50). Myelography can yield false negative results in cases of IVDE, primarily with lateral or foraminal extrusions, with these cases requiring CT or MRI (51).

Estimates of diagnostic accuracy of myelography for the diagnosis and site of IVDE have ranged from 72 to 99% (37, 45, 52, 53). Determination of lateralization is less accurate, especially when compared to CT or MRI. The reported sensitivity for lateral localization ranges from 49 to 83%, although the precision can be greatly improved with the combination of ventrodorsal and oblique views (15, 17, 30, 37, 45, 53, 54).

Myelography is an invasive procedure and is associated with some inherent risks, primarily temporary deterioration of neurologic status, and post-myelographic seizures (55, 56). Even though the incidence of post-myelographic seizures was as high as 21.4% in a study with 183 dogs (55), another study with 503 dogs found a incidence of only 3% (56). An important reason for this 7-fold difference relates to the volume of contrast medium administered. In the first study, the mean total volume of iohexol was 9.1 and 16.8 mL in dogs without and with seizures (55), whereas in the second study it was, 4.5 and 11.7 mL, in dogs without and with seizures, respectively (56). Importantly, the dose per kg was not associated with seizures in either study. It is therefore recommended to carefully assess the total volume required, and avoid using total volumes higher than 8 mL, even in large or giant dogs. Another key point to minimize the risk of seizures is the use of lumbar injections, since the risk of seizure was seven times higher with cerebellomedulary cistern injections in both studies (55, 56). Due to the risk of post-myelographic seizures, patients should be monitored in an ICU for at least 12 h post-myelography, which can significantly increase the expense associated with this procedure.

While myelography has limitations and has largely been superseded by cross-sectional imaging, it remains a reasonable imaging option for diagnosing IVDE. Importantly, in a study of 107 dogs assessing the choice of myelography or MRI for diagnosis of IVDE, patient outcomes using myelography were not inferior compared to MRI (57).

## Computed Tomography

Computed tomography (CT) is a very important modality in the diagnostic work-up of dogs and cats suspected of having IVDD (37, 52, 58, 59). Tomography by definition is simply the depiction of a section of the patient free from superimposition of overlaying structures (60). As conventional radiographs represent variations in tissue absorption of x-rays in a linear direction, physical structures are superimposed. Computed tomography transmits x-rays to detectors through the patient, around a single axis of rotation; the data is processed by a computer and creates the image as a slice, free of superimposition. Tissue physical density is measured relative to the density of water and assigned a numerical value called a CT number or Hounsfield unit (HU) (60).

There are 3 possible techniques to achieve a diagnosis of IVDE using CT: non-contrast CT, intravenous contrast CT (CT-angiography), and subarachnoid contrast CT (CT-myelography). CT-myelography requires ~25 to 50% of the volume of contrast medium compared to conventional myelography, and it is therefore much safer. In all of these CT modalities, transverse images can be reformatted into other imaging planes, primarily when using multidetector CTs which allow multiplanar reformatting in different spatial planes (37, 58, 61, 62). Advantages of CT relative to MRI include that it is more widely available, less costly and much faster. It is also possible to carry out CT studies routinely under sedation. Most, but not all, cases of IVDE can be diagnosed with non-contrast CT. In this context, CT can be used as a convenient, quick screening test for spinal cases where the neurosurgical caseload is high. For dogs in which non-contrast CT alone is insufficient, MRI or CT-myelography can then be utilized for a diagnosis. Median examination times in a study were 4, 8, and 32 min, for helical CT, conventional CT, and myelography, respectively (37). These times were achieved with a single slice helical CT. With newer systems ranging from 16 to 128 slices, scanning times are much shorter, often <1 min.

Most investigations relating to IVDD have been dedicated to non-contrast CT. The normal intervertebral disc is of uniform soft tissue opacity in CT images, with no visible distinction between the nucleus pulposus and annulus fibrosus (63). The spinal cord, cerebrospinal fluid, and meninges are of similar tissue physical density and cannot be clearly discriminated without the introduction of intra-thecal contrast media (64). This combination of structures is referred to as the thecal sac in plain CT images. Epidural fat surrounds the thecal sac and is less dense than soft tissue, so it appears darker gray. This difference in tissue density allows discrimination of the outer margins of the thecal sac. Calcified disc material is visible in non-contrast enhanced CT images because it has a higher physical (radiographic) density than adjacent soft tissues and fat (65).

**Figure 2:**
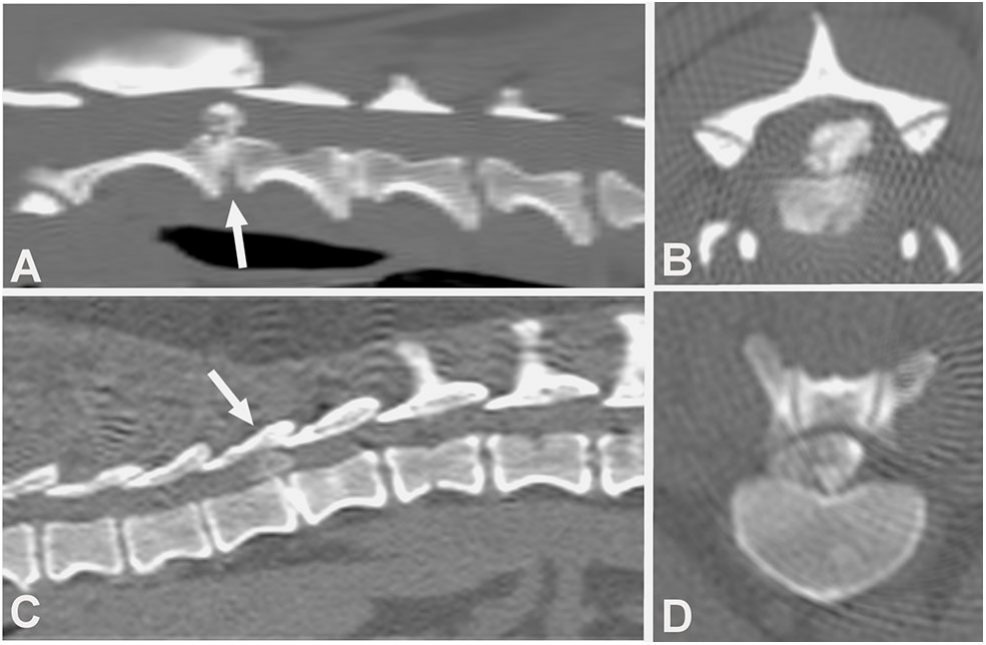
Computed tomographic (CT) images showing the appearance of mineralized intervertebral disc extrusion in the vertebral canal. **(A)** Sagittal reconstructed non-contrast CT image showing a hyperattenuating mass suggestive of extruded disc material into the vertebral canal between C2-3 (arrow). The intervertebral disc space also mineralization at C2-3. **(B)** Transverse non-contrast CT image showing a large hyperattenuating mass into the vertebral canal at the intervertebral disc level C2-3. **(C)** Sagittal reconstructed non-contrast CT image showing hyperattenuating material into the vertebral canal between T1-12 (arrow). **(D)** Transverse non-contrast CT image showing a large hyperattenuating mass disc extrusion occupying most of the vertebral canal at the intervertebral disc level T11-12.

The CT characteristics of acute IVDE include hyperattenuating material within the vertebral canal, loss of epidural fat, and distortion of the spinal cord (Figure 2). A case series of 23 surgically confirmed thoracolumbar IVDE categorized the CT patterns of herniation into 3 groups: acutely extruded mineralized nucleus pulposus; acute extrusion of nucleus pulposus with hemorrhage; and chronic mineralized nucleus pulposus (65). The acutely extruded, mineralized nucleus pulposus group had heterogenous, hyperattenuating (mean 219 HU) extradural masses causing severe spinal cord compression. The acute extrusion of nucleus pulposus with hemorrhage group was found to have herniated material causing less compression, extending over multiple vertebral spaces, and that was only slightly distinguishable from the spinal cord (mean 59 HU). Since the spinal cord itself cannot be directly visualized on plain CT, spinal cord compression in both groups of dogs was inferred from loss of the hypoattenuating epidural space. In the chronic, mineralized nucleus pulposus, the extruded material was described as extremely hyperattenuating (mean 745 HU) and more homogenous (65). In a study with 111 dogs with thoracolumbar IVDE, a 3–11% increase in mean certainty scores for correct diagnoses was found when multi-planar reconstruction (MPR) CT images were used (mainly oblique transverse and curved dorsal MPR views) compared to 2D CT images (61).

**Figure 3:**
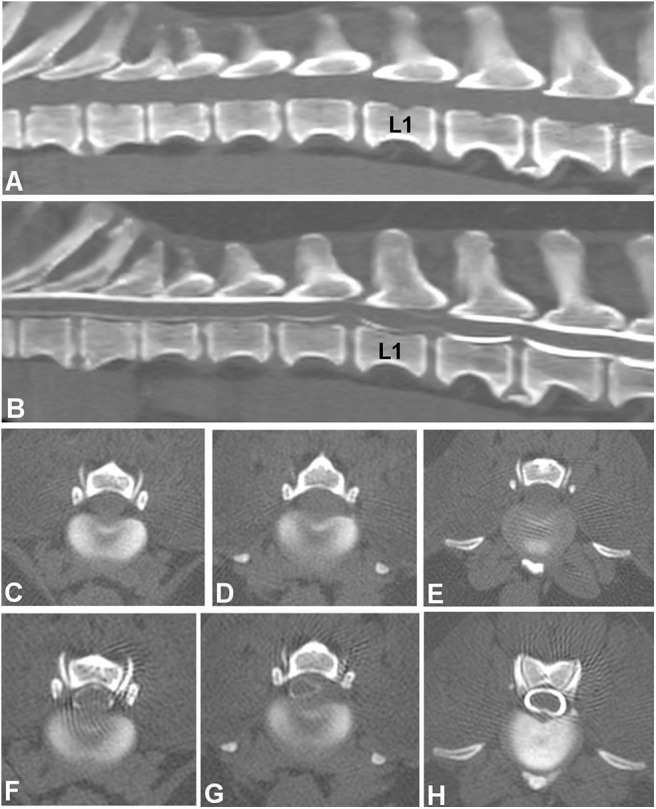
Images of a 10-year old female spayed Beagle presented with an acute onset of non-ambulatory paraparesis and spinal pain. **(A)** Sagittal non-contrast CT image revealing areas of spondylosis and no obvious compressive lesion. **(B)** CT-myelography shows areas of ventral subarachnoid space compression at T13-L1, L1-2, and L2-3. Thinning of dorsal and ventral contrast columns is noted between L1-2. **(C–E)** images show transverse non-contrast CT images representing T13-L1, L1-2, and L2-3 intervertebral levels, respectively. Peridural fat is lost at L1-2 level. **(F–H)** CT-myelographic images at levels T13-L1, L1-2, and L2-3. Ventral, lateral and dorsal spinal cord compressions are noted at L1-2. Surgical decompression and biopsy confirmed that the compressive material was extruded disc material.

Several studies compared the sensitivity of non-contrast CT to myelography and/or CT-myelography for the identification of IVDE in the thoracolumbar spine. A large retrospective study evaluated non-contrast CT and myelography in 182 dogs of varying breeds (58). Both methods had similar sensitivities, 81.8% for CT and 83.6% for myelography, though CT was better for chronically affected dogs (58). The same percentage of 81.8% was reported in another study using non-contrast CT (66). In this study, CT-myelography was required to confirm the diagnosis in the remaining 18.9% of cases (66). In a case series of 19 dogs, agreement with surgery was found in 94.7, 100, and 94.7% of cases using helical CT, conventional CT or myelography, respectively (37). In a prospective study using CT-myelography as the gold standard, non-contrast CT was found to be more sensitive than conventional myelography with a longitudinal and lateral localization accuracy of 91 and 94%, compared to 64 and 74% (Figure 3) (54).

A retrospective study suggested that CT-angiography was as diagnostic as CT-myelography in dogs with IVDE, with sensitivities of 97 and 94%, respectively (67). This finding was not corroborated by other studies. CT-angiography was evaluated in a prospective study and had the lowest sensitivity of all CT techniques (lower even than plain CT), at only 53% (52), whereas in another study it did not add any additional information compared to non-contrast CT (68). The diagnostic accuracy of CT for IVDH was 88–90% in two prospective studies where it was compared to MRI (69, 70).

A prospective study of 46 dogs with acute cervical and thoracolumbar myelopathies mimicking the diagnostic work-up of any acute spinal case, compared four modalities: non-contrast CT, CT-angiography, myelography, and CT-myelography, using as gold standard either surgery or necropsy (52). Almost 80% of dogs had extradural lesions and most had IVDE. Overall diagnostic sensitivity for all techniques was 66% for non-contrast CT, 53% for CT-angiography, 79% for myelography, and 97% for CT-myelography. Non-contrast CT had a sensitivity of 91% for IVDE, though it was not able to identify the associated spinal cord swelling in more severely affected dogs noted with other modalities. This led the authors suggest that CT-myelography should be the technique of choice for paralyzed patients to allow wider decompressive procedures (52).

A retrospective study of 555 dogs with thoracolumbar myelopathy investigated parameters that could be used to predict the need for additional imaging beyond non-contrast CT (68). The vast majority of the population had IVDE (94.6%, 525 dogs) and were chondrodystrophic (81.1%, 455 dogs), therefore the population studied was biased toward the likely diagnosis of IVDE. Additional imaging (either myelography, CT-myelography or MRI) was required in 17.4% of non-chondrodystrophic breeds, but in only 3.6% of Dachshunds. Timing of imaging was the second most important factor leading to additional imaging, with only 4.8% of dogs scanned during business hours requiring additional imaging. This number almost tripled (13.6%), when dogs were imaged out-of-hours. Patient preselection for CT or MRI explains this difference, as MRI was not available out-of-hours at the investigator's institution (68).

Based on all the available information, it is possible to conclude that non-contrast CT is as diagnostic as myelography for the localization of IVDE in chondrodystrophic dogs. It can be reliably used in the diagnostic investigation of cases suspected of having IVDD, primarily small breed, young dogs, emphasizing the point that case selection is key when electing a diagnostic work-up with non-contrast CT. The selection of non-contrast CT should also be undertaken with the acceptance that further imaging, either CT-myelography or MRI, might be required should no lesion or a lesion not matching the neurolocalization be identified (37, 52, 54, 59, 65, 68, 70, 71).

## Magnetic Resonance Imaging

MRI is considered the gold standard for diagnostic imaging in IVDD in both humans and veterinary patients. Manipulation of the various imaging characteristics of tissues allows the multiple anatomical structures within the vertebral column to be distinguished, including the supporting ligamentous structures, synovial joints, bone marrow, nerve roots, spinal cord parenchyma, cerebrospinal fluid, epidural fat, and the layers of the IVD (60). Images can be acquired in multiple planes without repositioning the patient, which is an advantage because image quality is always the same. However, the more planes, the longer the examination, a disadvantage compared to CT. Also, whereas myelography might be required in conjunction with CT for the diagnosis of IVDD, this is not necessary with MRI because of the ability to alter tissue contrast by applying different acquisition sequences. Thus, the risks associated with myelography are avoided. A key point is that IVDE can go undetected on myelography or CT, but the likelihood of a false negative result with MRI is much lower (30, 69, 70, 72).

There is substantial variation in MRI quality in veterinary practice with both low and high field MRIs available. Due to upfront and maintenance costs, low-field (0.2–0.4 T) units currently greatly outnumber high-field ones, primarily in private referral practices (73). While low field units offer diagnostic quality in the vast majority of studies, the compact magnet design of some veterinary scanners may not allow scanning or have significant quality issues in the caudal cervical or cranial thoracic spine of very large or giant breed dogs (73). Similarly, these scanners have a small maximal field of view, that may require patient repositioning when examining larger regions, thus making it more time consuming than with high field MRI systems. Spinal studies in very small dogs or cats (<3 kg) can also be of questionable diagnostic quality, as many low-field systems cannot have slices thinner than 3 mm. Enlarging the field of view (FOV), increasing slice thickness, and increasing the number of acquisitions will increase the signal to noise ratio (which means improved image quality), but these adjustments reduce image resolution and prolong anesthesia and magnet time (59). High field MRI units typically range from 1.0 to 3.0 T, with the most popular unit in veterinary hospitals being the 1.5 T. In general, the higher the field strength, the higher the signal to noise ratio and the faster the imaging times, to a limit (71).

As the diagnostic quality and accuracy of MRI for the diagnosis of IVDD are dependent on several technical aspects, a brief overview of these factors is warranted. For a comprehensive review of technical aspects, the reader is referred to other sources (74–76).

## Positioning

Patients are typically positioned in dorsal recumbency. It is extremely important to achieve a straight spinal alignment to allow comparison of multiple intervertebral sites on sagittal images. Sandbags and foam wedges are very useful for this purpose (71). The high prevalence of vertebral body malformation, leading to kyphosis and scoliosis in brachycephalic screw-tailed dogs, like the French bulldog, can make straight spinal alignment impossible to be achieved (77).

## Acquisition Planes

The sagittal plane is used initially to localize specific intervertebral regions to be investigated with transverse slices. The FOV of the sagittal plane in low field MRIs can be small, so determination of the affected intervertebral disc(s) level can be challenging in the thoracolumbar region without reliable cranial and caudal vertebral references. A strategy commonly used is the identification of celiac and mesenteric arteries. In one study the celiac artery and mesenteric arteries were ventral to the first lumbar vertebra in 71 and 97% of dogs, respectively (78). In another study where they were under the body of L1 in only 59.6% of dogs, with an additional 11.2 and 20.2% under the intervertebral discs T13-L1 and L1-2, respectively (79). As such, their location is variable and they do not represent an accurate vertebral landmark. The dorsal plane can be very useful in these instances since the ribs and transverse processes can be easily identified. In cases where dorsal images are not available, sagittal (parasagittal) images can be used (79). Rib articulations and lumbar transverse processes can be easily distinguished based on different shapes and heights relative to the vertebral canal (79). The transverse plane is essential for circumferential assessment of spinal cord compression and lateralization. Selection of transverse sections is typically based on the identification of compressive sites on sagittal images. The reliability of sagittal T2-weighted (T2W) images alone for the determination of compressive disc lesions was determined to range from 81.4 to 89.0%. This means that without acquiring transverse images, a compressive lesion would have been missed in 11–18.6% of dogs, with almost 10% being considered clinically relevant (80). It is therefore recommended to always obtain transverse images at least encompassing the disc spaces immediately cranial and caudal to the supposedly affected disc, or the entire spinal region if multiple areas appear abnormal (80).

## Image Sequences

**Figure 4:**
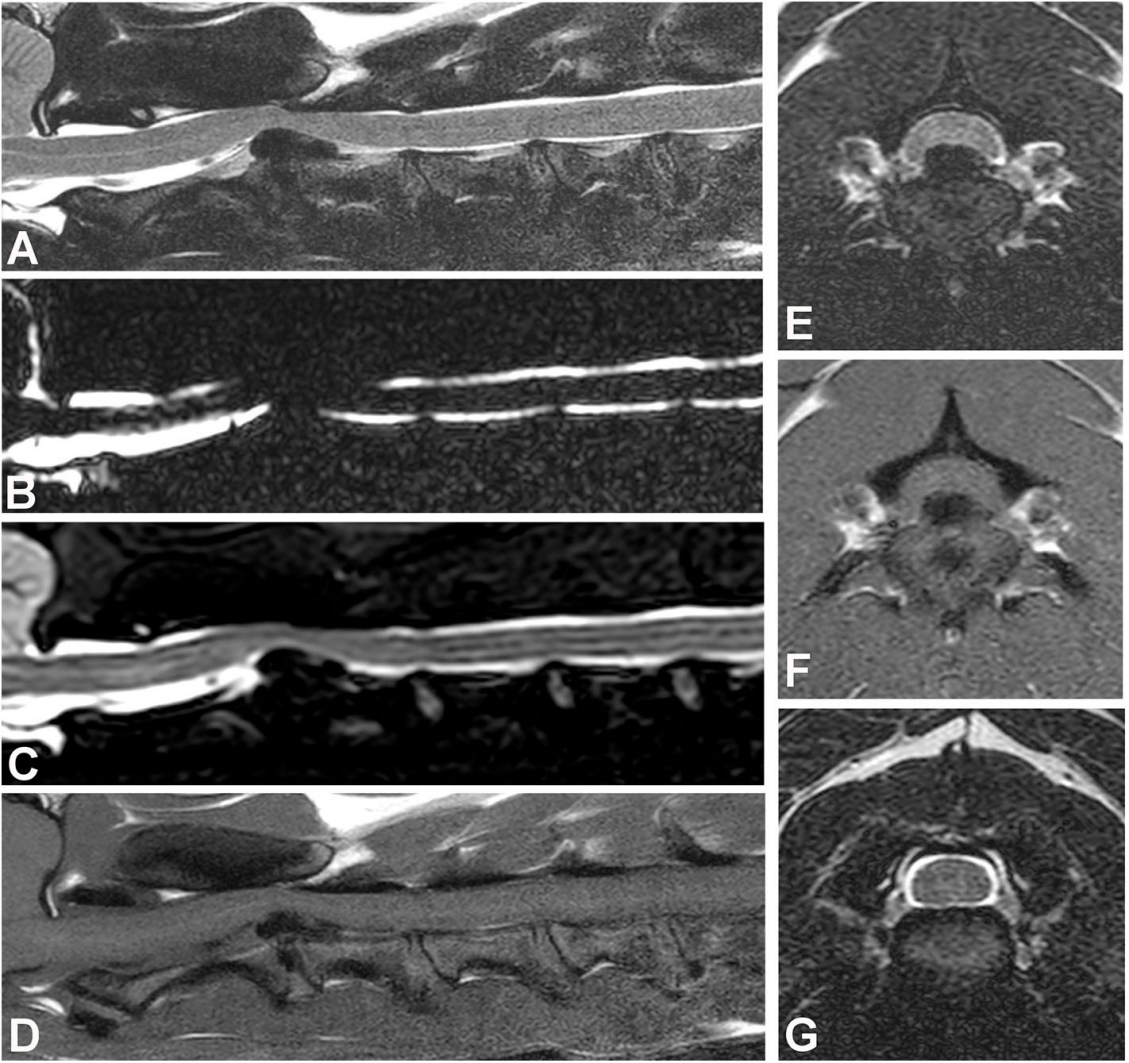
Images of a dog with cervical intervertebral disc extrusion. **(A)** Sagittal T2W image. **(B)** Sagittal HASTE image. **(C)** Sagittal STIR image. **(D)** Sagittal T1W image. All sagittal images show ventral extradural compression in the ventral aspect of the vertebral canal at the intervertebral level C2-3. Note how the compressive material is hypointense in all images, though image resolution varies with different sequences. The signal of intervertebral discs *in situ* also varies in all sequences. **(E)** Transverse T2W image showing a large hypointense mass causing spinal cord compression at the C2-3 level. **(F)** Transverse T1W image showing the hypointense compressive material at C2-3. **(G)** Transverse T2W image showing the C3-4 level with no spinal cord compression.

While there are variations regarding preferences for sequences and planes, it is generally agreed that T2W and T1W images in both sagittal and transverse plane should be routine (Figure 4). The importance of transverse T1W images is typically minimized, although they are very useful to help distinguish extradural compression caused by extruded nuclear material, typically hypointense, from soft tissue masses and hemorrhage. Similarly, post-contrast T1W images, although not routinely used, increased the ability to determine the site and side of IVDE in one study (30). Ultrafast heavily T2W, short T1 inversion recovery (STIR), and gradient echo sequences (GRE, also known as T2^*^ or fast field echo—FFE) are also routinely acquired (Figure 4). A low-field MRI study looked at sagittal STIR images compared to sagittal T2W and found no difference (81). However, the parallel evaluation of the paired sagittal T2W and STIR series yielded a higher sensitivity than using either sagittal screening series in isolation (81). Heavily T2W images, also called “myelo-MRI” or HASTE (Half-Fourier acquisition single-shot turbo spin echo) images, can be used to highlight the cerebrospinal fluid signal and rapidly identify an area of interest (Figure 4). They are very fast sequences, so a valid addition in MRI spinal protocols, though two studies showed that they are not as reliable as sagittal T2W images for identification of compressive IVDD lesions (80, 82). They are useful in distinguishing acute from chronic cases of IVDH and as a predictor of progressive myelomalacia.

## MRI of Intervertebral Disc Degeneration

MRI allows clear visualization of the intervertebral disc. The nucleus pulposus and annulus fibrosus are best appreciated on T2W images. In the healthy intervertebral disc, both the nucleus pulposus and the inner part of the annulus fibrosus are hyperintense on T2W images, appearing as a hyperintense ellipsoid area on sagittal images (83). The degree of brightness of the nucleus pulposus in the T2 signal correlates with the proteoglycan concentration but not with water or collagen concentration (84). Annular and nuclear disc material cannot be distinguished in T2W images of degenerative IVDs due to biochemical changes in the extracellular matrix of the nucleus. A lower water content and a shift of proteoglycan composition of the nucleus pulposus results in a lower signal intensity in T2W images, making annular and nuclear material iso-intense to each other (84, 85). It has been long proposed that IVDE results from IVD degeneration (4). It is important to emphasize that disc degeneration is a very common finding in clinically normal dogs, and *per se*, does not lead to clinical signs, except in uncommon cases of discogenic spinal pain (71, 86).

**Figure 5:**
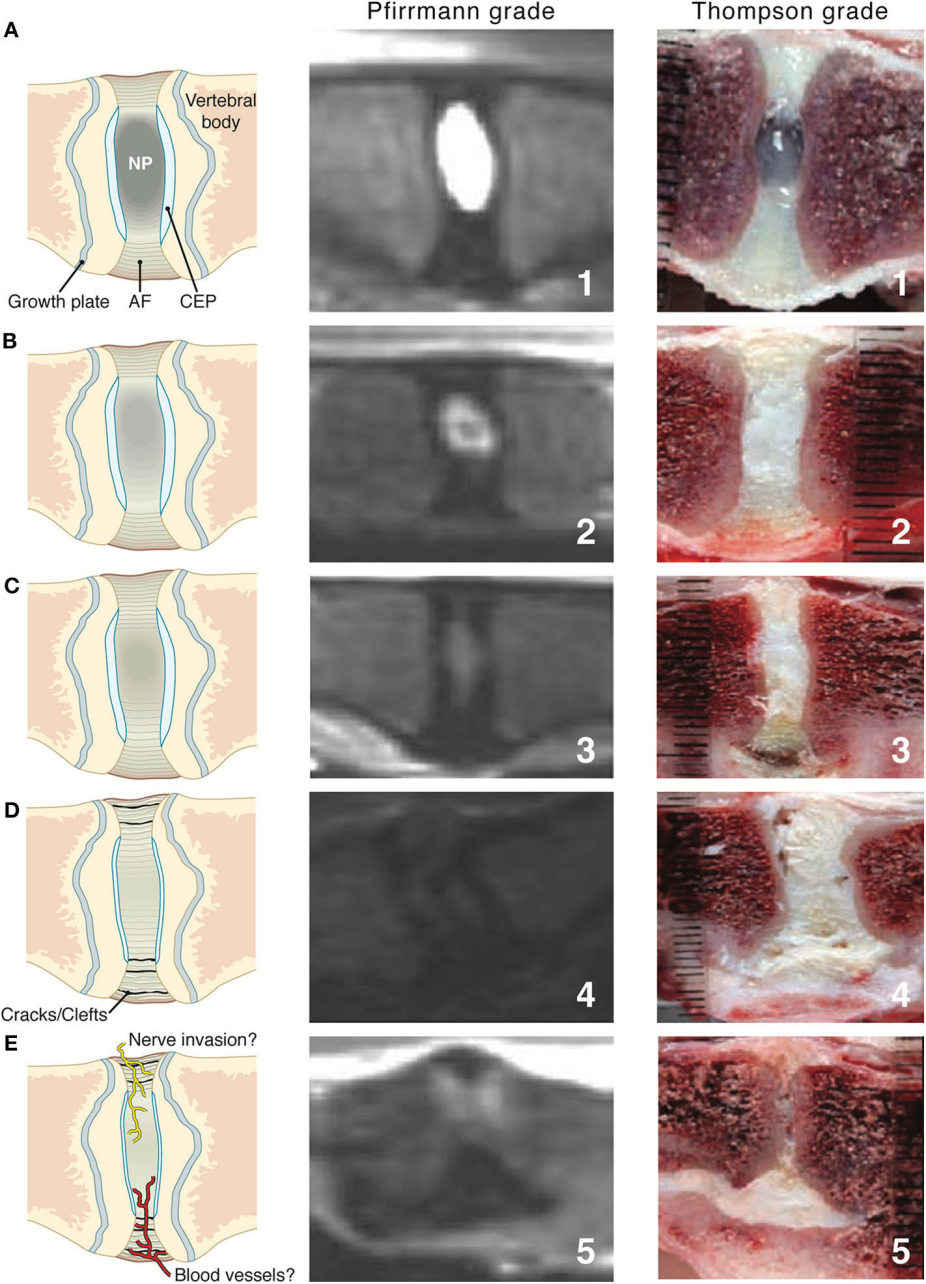
Intervertebral disc (IVD) maturation from young to early and late stage IVDD where the first column shows illustrative representations throughout the stages **(A–E)**, middle column shows Pfirrmann grading via T2W MR images and last column showing Thompson grading of canine IVD. AF, annulus fibrosus; CEP, cartilaginous end plate; NP, nucleus pulposus. Pfirrmann grade and Thompson grade images adapted with permission from Bergknut et al. (90) and Thompson et al. (91).

In human medicine, the Pfirrmann system is the most widely used system for grading IVD degeneration on the basis of sagittal T2W MRI findings (87). It is based on a system for grading gross pathological changes in IVDs proposed by Thompson et al., which is the most commonly used criterion-referenced standard in human medicine (88). The Pfirrmann system has been validated in dogs, showing good correlation with age and chondrodystrophic breeds (89), as well as the Thompson macroscopic grading scheme for disc degeneration (Figure 5) (20, 90). A study looked at a modified Pfirrmann grading system using images from a novel T1 weighted FFE-sequence images compared with T2W images (92). It was concluded that T2W images should remain the sequence of choice to grade IVD degeneration (92).

The initiating factor for IVDD in humans is thought to be a loss of diffusional capacity of the vertebral endplate blood vessels that provide nutrition for the nucleus pulposus (93).

Endplate changes have also been thought to be associated with canine IVDD (94, 95), and were recently investigated in dogs with and without IVDD (96). The most common abnormal endplate change was hyperintensity on T1W and T2W images (97.2% of dogs). These changes were significantly associated with the presence of IVDD in the adjacent disc. Dogs with vertebral endplate changes anywhere in the vertebral column were not, however, more likely than dogs without vertebral endplate to have IVDD (96).

## MRI Findings in Intervertebral Disc Extrusion

MR imaging features of IVDE include extradural compression of the spinal cord centered over or near the intervertebral disc space. This mass effect caused by the extruded material causes compression and/or displacement of the spinal cord, seen on T2W images as displacement or loss of the hyperintense signal associated with subarachnoid and epidural spaces. Extruded nucleus pulposus is typically identified as a hypointense mass within the epidural space on T1W and T2W images (Figure 4). It can be characterized as dispersed if it is not associated with the affected intervertebral space and spread throughout the epidural space, or non-dispersed if it remains in contact with the affected IVD (94). The MRI features of IVDE in cats are similar to dogs, with the difference that most reported cats have had disc extrusions in the lumbar vertebral column, as opposed to the caudal thoracic and thoracolumbar (T12-13, T13-L1) as seen in small breed dogs (21, 97–99).

**Figure 6:**
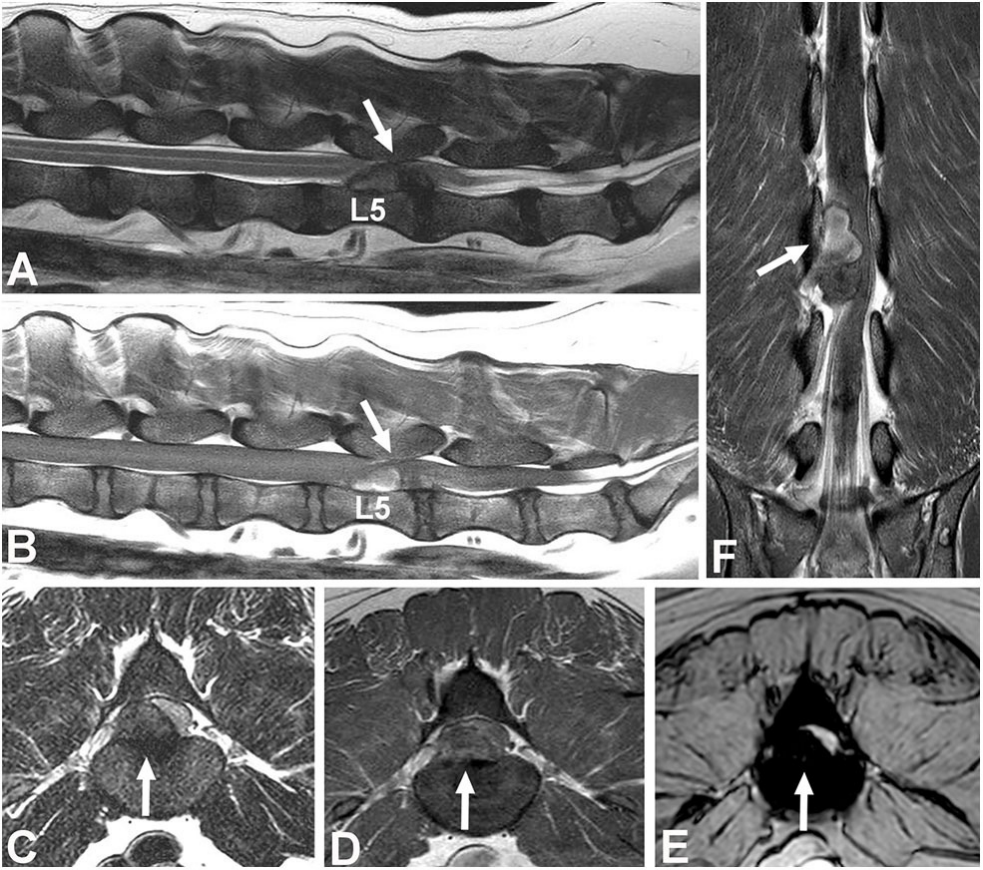
Images of a female spayed, 6-year-old, mixed breed dog with an acute onset of paraparesis, spinal pain, and fecal and urinary incontinence. **(A)** Sagittal T2W image showing a large mass with mixed signal intensity between L5-6 (arrow). **(B)** Sagittal T1W image showing that the cranial aspect of the mass is hyperintense (arrow). **(C)** Transverse T2 image showing severe spinal cord compression caused by a large hypointense mass between L5-6 (arrow). **(D)** Transverse T1W contrast-enhanced image showing mild heterogeneous contrast enhancement of the hypointense lesion. **(E)** Gradient echo image showing marked hypointensity. **(F)** Dorsal T1W contrast-enhanced image showing contrast enhancement of the cranial aspect of the compressive lesion. Note the length of the compressive lesion. Surgical decompression and biopsy confirmed that the compressive material was extruded intervertebral disc with hemorrhage.

Epidural hemorrhage associated with IVDE can result in a wide range of signal intensities, including signal void, thus a diagnosis of IVDE should not rely on one pattern of signal intensity (Figure 6) (100, 101). Gradient echo sequences can confirm the presence of hemorrhage.

The degree of spinal cord compression can be categorized based on morphologic compression scales (102). A common categorization is based on the percentage of reduction in spinal cord diameter, graded as mild (<25%), moderate (25 to 50%), or severe (>50%) (102, 103). Morphometric estimates of cross-sectional area of either spinal cord or extruded material offer even more precise information (104–106). The degree of spinal cord compression seen on transverse MR images in cases of IVDE was associated with the severity of pre-operative neurological signs in the cervical spine (107), but not in the thoracolumbar region (94, 108, 109).

Contrast enhancement of extradural compressive material can be seen in dogs with IVDH, primarily in dogs with IVDE (60%) compared with 16% of dogs with IVDP (110, 111). Various patterns of enhancement can be seen, including homogeneous, heterogeneous, and even peripheral enhancement patterns (110). These findings can be misinterpreted as a granulomatous or neoplastic lesion, thus it is important to be aware of these contrast patterns to avoid misdiagnosing IVDE as another condition (71). Meningeal enhancement adjacent to the extruded disc material was also noted in up to 40% of dogs (111).

Paravertebral muscle signal changes were seen in 36% of dogs with IVDE or ANNPE in one study. These changes are characterized by an edematous pattern that is hyperintense on T2W and iso- or hypointense on T1W sequences and are best visualized on T2W fat-suppressed sequences. No signal void is seen on T2^*^W GRE and a variable degree of contrast enhancement is seen in 45% of dogs. Paravertebral signal alterations are more commonly seen in disc extrusions caudal from L1 and in animals with a more severe neurological grade. Histopathology typically does not reveal specific abnormalities and the underlying pathomechanism might be related to ischemia, muscle spasm or denervation edema (112).

**Figure 7:**
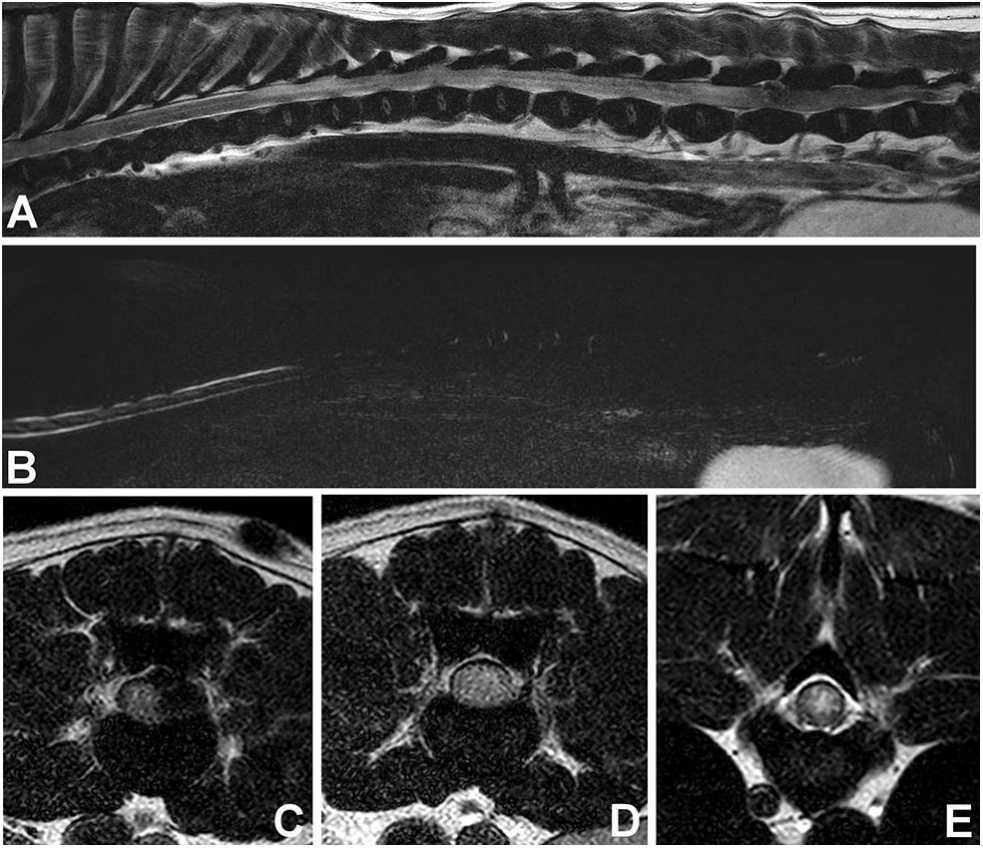
Images of female spayed, 13-year-old Cocker Spaniel with an acute onset of paraplegia. **(A)** Sagittal T2W image showing a large area of diffuse spinal cord hyperintensity. **(B)** Sagittal HASTE image showing diffuse spinal cord swelling. **(C)** Transverse T2W image at the L5-6 level showing lateralized spinal cord compression and spinal cord hyperintensity. **(D)** Transverse T2W image at the L3-4 level showing diffuse spinal cord hyperintensity and apparent swelling. **(E)** Transverse T2W image at T9-10 level showing centrally located spinal cord hyperintensity. Myelomalacia and intervertebral disc extrusion at L5-6 were confirmed at post-mortem examination.

In dogs with acute thoracolumbar disc extrusion, areas of spinal cord hyperintensity on T2W images can be observed (Figure 7). These spinal cord hyperintensities have been shown to correlate with the severity of neurologic signs at presentation (94, 109, 113). The specific pathologic processes associated with hyperintensity on T2W images are not fully known, but have been thought to involve necrosis, myelomalacia, intramedullary hemorrhage, inflammation, and edema (114–116). The prognostic value of spinal cord hyperintensity on T2W images is subject of considerable controversy, with newer studies (117, 118), questioning older findings (109, 113, 119). Interestingly, a recent low-field MRI study of IVDE found spinal cord hyperintensity on T2W images in 18% of dogs in the preoperative period. MRI was repeated after decompressive surgery (immediately after surgery in most dogs) and the number of dogs with hyperintensity almost doubled (34%) (105). The relationship between spinal cord hyperintensity and prognosis of IVDD can be found in the article “Prognostic factors in acute spinal cord injury” in this issue.

Two prospective studies compared non-contrast CT (helical multislice systems) vs. MRI (1.0 T systems) for the diagnosis of thoracolumbar IVDH, using surgery as the gold standard. The first study had 44 dogs and found that the sensitivity of MRI was 98.5%, compared with 88.6% for CT for identifying the site of IVDH (69). In another study with 40 dogs with confirmed IVDE, a lesion was detected using MRI in all dogs, whereas CT did not allow identification of a lesion in 4 out of 40 dogs (10%) (70). It can be concluded based on these studies that MR imaging has a sensitivity approximately 10% higher than non-contrast CT for the diagnosis of thoracolumbar IVDE. This 10% difference was also seen when MRI was prospectively compared to myelography (30). The overall diagnostic accuracy of MRI in dogs with IVDE is therefore between 98.5 and 100% (30, 69, 70, 72).

While MRI has been reported to be more accurate than CT in discriminating IVDE from IVDP, this distinction can still be difficult based on MRI (69). In order to assist with this differentiation, criteria have been proposed and are discussed in the IVDP section below.

## MRI and Surgery

MRI findings have been compared to actual surgical findings by assessing the cranio-caudal extent of extruded disc material using calipers intraoperatively (72). In two thirds of dogs, authors observed 100% agreement between MR imaging and surgical findings, but only 50% agreement in the remaining third of dogs (72). A prospective study compared surgical planning using MRI and CT in a population of 40 dogs with IVDE. Hemilaminectomy planning varied in about 50% of cases between MRI and CT (43.5–66.6%). In all cases where planning differed, a significantly larger hemilaminectomy defect (a greater number of articular facets removed) was planned using MRI compared to CT (70). MRI is also a very useful modality to investigate patients in the post-operative period (105, 120–122). MR imaging can differentiate residual extruded nucleus from hemorrhage, based on signal intensity and gradient echo characteristics (101, 120). Gelatin sponges can also be easily differentiated from residual disc material based on their sharply demarcated shape and hyperintensity on T2W images (120). MRI was also very accurate (100%) for identification of late recurrent disc extrusion either at the same or different site (mean interval between initial surgery and recurrence 404.5 days) in a study comparing it to myelography (122).

A recent prospective low-field MR study evaluated the agreement between surgeon's perception of spinal cord decompression and residual disc on postoperative MR images acquired immediately after surgery in most cases (105). In most instances in which the MRI results differed from the surgeon's perception, the degree of surgical decompression was perceived as satisfactory by the surgeon, but was revealed as unsatisfactory via MRI. This was observed primarily in large disc extrusions (105). The clinical outcome of those dogs with unsatisfactory spinal cord decompression was worse than those with adequate spinal cord decompression (105). Another prospective high-field MRI study found residual spinal cord compression in 44% of dogs treated with mini-hemilaminectomy (120). The median percentage of residual material was 7.7% of the preoperative volume, and the mean degree of residual spinal cord compression was 10.9%, compared with 37% before surgery. The volume of residual disc in this study did not appear to have an influence on outcome (120).

## MRI Features of Progressive Myelomalacia

Progressive myelomalacia (PMM) is one of the worst complications seen in dogs with IVDE. It is reported to occur in ~10–17.5% of paraplegic dogs with absent pain perception (123, 124), and as high as 33% in French Bulldogs (125). Even though it is a clinical diagnosis in many cases, it is important to recognize its MRI features. MR imaging of PMM shows severe spinal cord swelling, more easily appreciated in the heavily T2 sequences (HASTE, myelo-MRI), along with diffuse parenchymal hyperintensity on T2W images over several spinal cord segments (Figure 7). On transverse T2W images, the hyperintensity is centered in the gray matter. FLAIR images will also reveal diffuse hyperintensity, whereas GRE images will show diffuse hypointensity (126, 127). Since myelomalacia is a form of hemorrhagic necrosis of the spinal cord, depending on when MRI studies are performed, it is possible to also observe hypointensity in the T2W images (126). A study proposed that diffuse spinal cord hyperintensity 6 times longer than the body of L2 vertebral body was suggestive of PMM (126), however this MRI pattern was seen in only 45% of dogs in another study (128). Others suggested that an area of hyperintensity 4.57 times longer than the body of L2, or of loss of cerebrospinal fluid signal on HASTE (MR-myelo) sequences 7.4 times longer than the body of L2 were at higher risk for PMM (Figure 7) (129, 130). Importantly, dogs can develop PMM without the presence of spinal cord hyperintensity on MRI (129).

## Intervertebral Disc Protrusion

In contrast to IVDE, the degenerative process in IVDP is much slower, with concurrent changes in the annulus fibrosus and nucleus pulposus. The annulus progressively loses its structural integrity, which allows the nucleus pulposus to move dorsally into the weakened annulus fibrosus. This will cause gradual protrusion of the dorsal annulus fibrosus into the vertebral canal, ultimately resulting in chronic progressive spinal cord compression. The pathophysiology, classification and clinical presentation of animals with IVDP is discussed in more detail in a companion article of this issue by Fenn et al.

Although IVDP is a common spinal disorder (131), only a few studies have focused on the specific imaging characteristics of this type of IVDD (46, 132). There are, however, indications that thoracolumbar IVDE and IVDP might be associated with differences in recommended surgical techniques, surgical complications, and outcomes after surgical and medical management (46, 133–135). Differentiation between IVDD sub-types is therefore clinically important.

Similar to IVDE, spinal radiographs cannot be used to confirm a diagnosis of IVDP. Common radiographic abnormalities seen in dogs with IVDP include vertebral endplate sclerosis (67%), and spondylosis deformans (47%) with narrowing of the intervertebral disc space occurring less commonly (25%) (46). Although spondylosis deformans has been associated with the presence of IVDP, this radiographic finding is also commonly observed in clinically normal animals. Spondylosis deformans can therefore not be considered a reliable indicator for IVDP or IVDD in general (136). Additionally, survey radiographs can be normal in dogs with IVDP but are often performed to look for other diseases with similar clinical presentation, such as vertebral neoplasia.

Until two decades ago, myelography was probably the most commonly used imaging modality to diagnose spinal conditions in veterinary medicine (16). Myelographic criteria have been reported to differentiate between IVDE and IVDP (46). The criteria for IVDP were (1) thinning and dorsal or dorsolateral deviation of the contrast columns, (2) thinning of the contrast columns is mild, (3) thinning of the contrast columns is focal and centered on the cranial and caudal boundaries of the affected disc, and (4) there is a symmetrical distribution of contrast column thinning cranial and caudal to the affected disc (Figure 1). This compares to the myelographic criteria for IVDE previously listed in the IVDE section. It was reported that application of these myelographic criteria allowed reliable differentiation between IVDE and IVDP when compared to intraoperative findings (46). For reasons outlined above including diagnostic limitations and procedural risks, myelography has now largely been replaced by more advanced diagnostic imaging modalities, such as CT and MRI (71).

Although CT is widely used to diagnose acute and chronic IVDE, there are no studies that have specifically focused on the use of CT to diagnose IVDP. The herniated material in IVDP is not mineralized and is therefore difficult to visualize on non-contrast CT. Spinal cord compression can further not be directly visualized on non-contrast CT and it is therefore likely that it cannot reliably be used to obtain a diagnosis of IVDP (59). Although fibrotic disc protrusions can occasionally be seen on CT, it is important to consider that a large number of dogs with IVDP have multiple affected intervertebral discs and that IVDH can also occur in dogs without clinical signs, further complicating the interpretation of CT findings in this population. While non-contrast CT failed to identify any of 16 cases of non-mineralized IVDP in one study, CT-myelography was able to diagnosis all of these cases (52). However, CT-myelography is associated with similar safety limitations as myelography. The CT-myelographic appearance of IVDP is typically characterized by a ventral extradural spinal cord compression with soft tissue density characteristics, overlying and not exceeding the intervertebral disc space. Chronic spinal cord compression can also result in irreversible spinal cord pathology and spinal cord atrophy (137). A circumferential widening of the subarachnoid space with a more triangular shaped spinal cord has been suggested to represent spinal cord atrophy (138). Although this is considered a negative prognostic indicator in human medicine, the prognostic role of this imaging finding is currently unclear in dogs with chronic spinal cord compressions (139).

**Figure 8:**
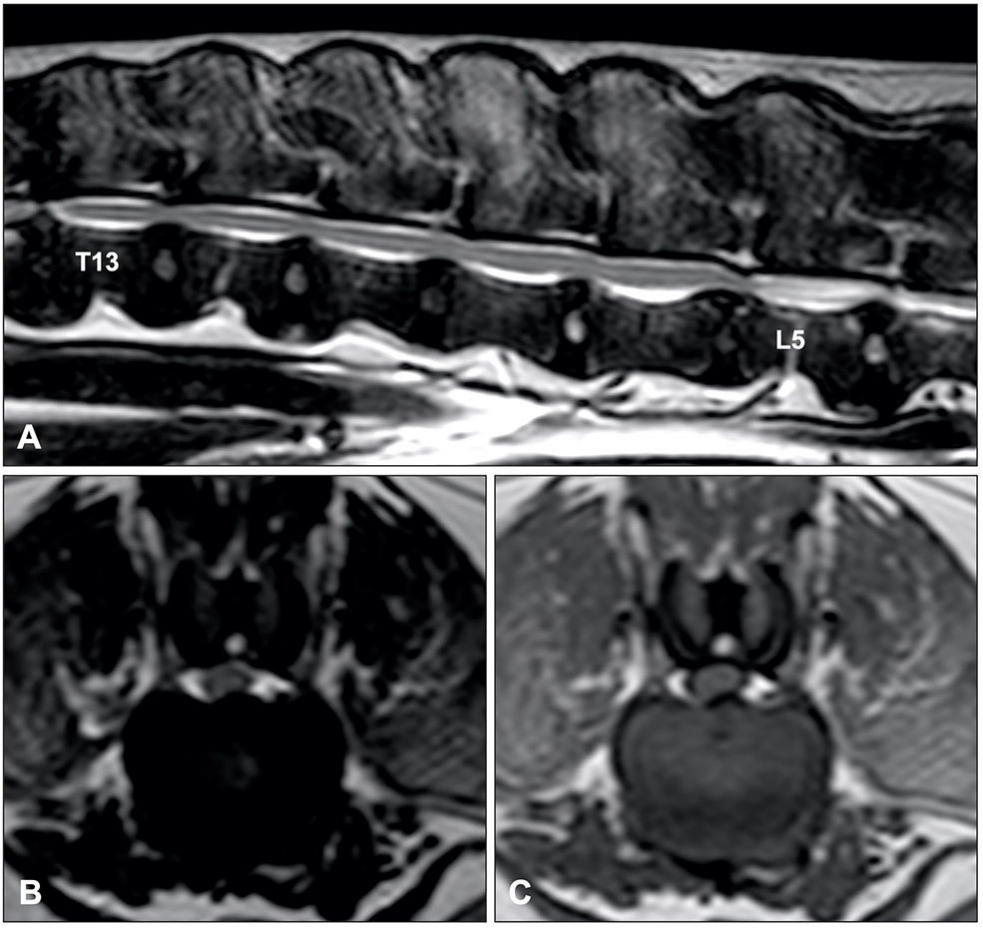
Sagittal T2-weighted **(A)**, and transverse T2-weighted **(B)** and T1-weighted MR **(C)** images of a 10-year old Dalmatian with multiple IVDPs between T13 and L6. The nucleus pulposus of most intervertebral discs has maintained some T2-weighted hyperintense signal, multiple discs are affected, intervertebral disk herniations are contained within the boundaries of the intervertebral disc space, and have resulted in midline spinal cord compression.

MRI has become the imaging modality of choice for most spinal disorders in veterinary medicine (71). Although the MRI characteristics of IVDD have extensively been reported, only a few studies have focused on IVDP (132, 140). One study identified four MRI variables that could be considered independent predictors of thoracolumbar IVDE vs. IVDP (Figure 8) (132):

- Midline instead of lateralized intervertebral disc herniation was associated with a diagnosis of IVDP
- Partial instead of complete loss of the hyperintense signal of the nucleus pulposus was associated with a diagnosis of IVDP
- The presence of a single intervertebral disc herniation instead of multiple intervertebral disc herniations was associated with IVDE
- Herniated material dispersed beyond the boundaries of the intervertebral disc space was associated with a diagnosis of IVDE.

In a later study by the same group, using these four criteria significantly improved diagnostic accuracy and interobserver agreement of MRI to differentiate thoracolumbar IVDD subtypes (140). It is currently unknown if these proposed MRI variables are also useful for cervical IVDH. It is worth mentioning that IVDP can be lateralized in both cervical and thoracolumbar regions.

**Figure 9:**
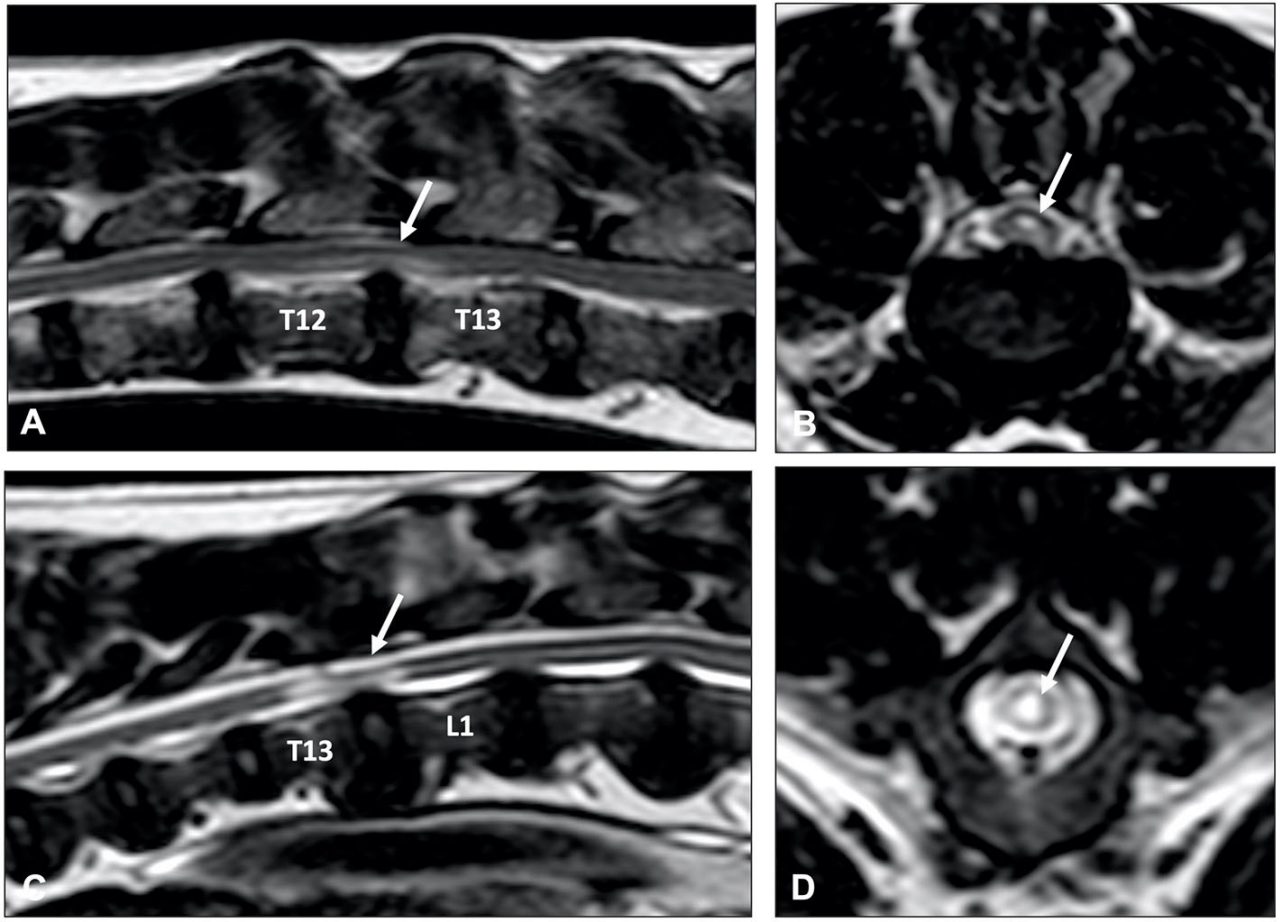
Sagittal **(A,C)** and transverse **(B,D)** T2-weighted MR images of a 9-year old Labrador retriever **(A,B)** and an 11-year old Poodle **(C,D)** with thoracolumbar IVDP. A poorly demarcated, diffuse intraparenchymal hyperintensity is seen overlying the affected intervertebral disc (arrow) in the Labrador retriever, while a well-demarcated and bright intraparenchymal hyperintensity is seen in the Poodle (arrow). It is currently unclear if this difference in intraparenchymal intensity characteristics is associated with differences in clinical presentation or outcome.

A common MRI finding in animals with chronic intervertebral disc herniations is focal T2W intraparenchymal intensity changes at the site of spinal cord compression (132). As previously suggested, the exact meaning of these abnormalities is unclear, but intraparenchymal intensity changes are likely to represent a wide spectrum of reversible and potentially irreversible pathological changes, such as edema, gliosis, and malacia (141). Although it has been suggested that intraparenchymal hyperintensity can aid in differentiation of clinically relevant from irrelevant disc-associated spinal cord compressions (104, 142), it is currently unclear if specific subtypes of intraparenchymal intensity changes can be used as prognostic indicators (Figure 9) (143). The high sensitivity of MRI can also complicate interpretation of MRI studies as intervertebral disc degeneration, herniation and even spinal cord compression can be observed on MRI studies of clinically normal, especially older, dogs (71, 104, 144). This underlines that abnormalities observed on MRI studies should always be correlated with results of thorough clinical and neurologic examinations, and that accurate interpretation of MRI studies requires experience and expertise.

Intervertebral disc protrusion can also be a prominent component of more complex and multifactorial neurological syndromes, such as degenerative lumbosacral stenosis and disc-associated cervical spondylomyelopathy (8, 143). Although a detailed description of these neurological syndromes is beyond the scope of this article and strict diagnostic criteria are lacking, it is common for dogs with these neurological syndromes to have a combination of bony and soft-tissue abnormalities contributing to vertebral canal stenosis. Additional abnormalities that can be observed on diagnostic imaging studies of dogs with degenerative lumbosacral stenosis include ligamentum flavum hypertrophy, articular process hypertrophy, telescoping of the sacral dorsal lamina, transitional vertebra, osteochondrosis, and vertebral malalignment (8). Additional abnormalities that can be seen in dogs with disc-associated cervical spondylomyelopathy include ligamentum flavum hypertrophy, craniodorsal vertebral tilting, abnormally shaped vertebral body, funnel shaped vertebral canal and articular process hypertrophy (143).

## Herniation of None or Minimally Degenerative Nucleus Pulposus Extrusion

Since MRI has become more widely available in veterinary medicine, additional types of IVDH have been recognized, which are characterized by sudden herniation of non-degenerative or minimally degenerative nucleus pulposus. The two most common types of extrusion of non-degenerative or minimally degenerative nucleus pulposus are acute non-compressive nucleus pulposus extrusion (ANNPE) and hydrated nucleus pulposus extrusion (HNPE) (145). Another type of IVDH that can be associated with herniation of non-degenerative nucleus pulposus is intradural/intramedullary intervertebral disc extrusion (IIVDE). It should however be noted that IIVDE has also been reported after IVDE (146). In contrast to IVDE and IVDP, herniation associated with ANNPE and HNPE occurs without advanced degeneration and dehydration of the intervertebral disc. Acute herniation of non-degenerative, well-hydrated nucleus pulposus material is predominantly associated with contusive spinal cord injury and has an uncertain role for sustained spinal cord compression. Acute non-compressive nucleus pulposus extrusion and HNPE are therefore associated with distinct pathophysiology, clinical presentation, diagnostic imaging findings, and recommended treatment options compared to that of compressive IVDH (145). The classification, terminology and clinical presentation of different types of IVDH are discussed in detail in a companion article in this research topic by Fenn et al. (“Canine Intervertebral Disc Disease: The Current State of Knowledge”). A diagnosis of ANNPE, HNPE, and IIVDE is based on a combination of compatible clinical signs and diagnostic imaging findings. Magnetic resonance imaging is considered the imaging modality of choice for ANNPE and HNPE, while CT-myelography has been considered particularly sensitive for diagnosis of IIVDE (6, 147, 148).

## Acute Non-Compressive Nucleus Pulposus Extrusion

Acute non-compressive nucleus pulposus extrusion is characterized by a sudden extrusion of non- or minimally degenerative nucleus pulposus without residual spinal cord compression. This condition has been reported with various terms over the years, namely Hansen Type III IVD herniation (even though Hansen did not report it), traumatic IVD extrusion, IVD explosion, missile discs, and high-velocity low-volume IVD extrusion. Affected animals typically present with a peracute onset of possibly strongly lateralized clinical signs, often during strenuous exercise or trauma. Severity of clinical signs should not progress after the initial 24 h and sustained spinal hyperesthesia should not be present.

**Figure 10:**
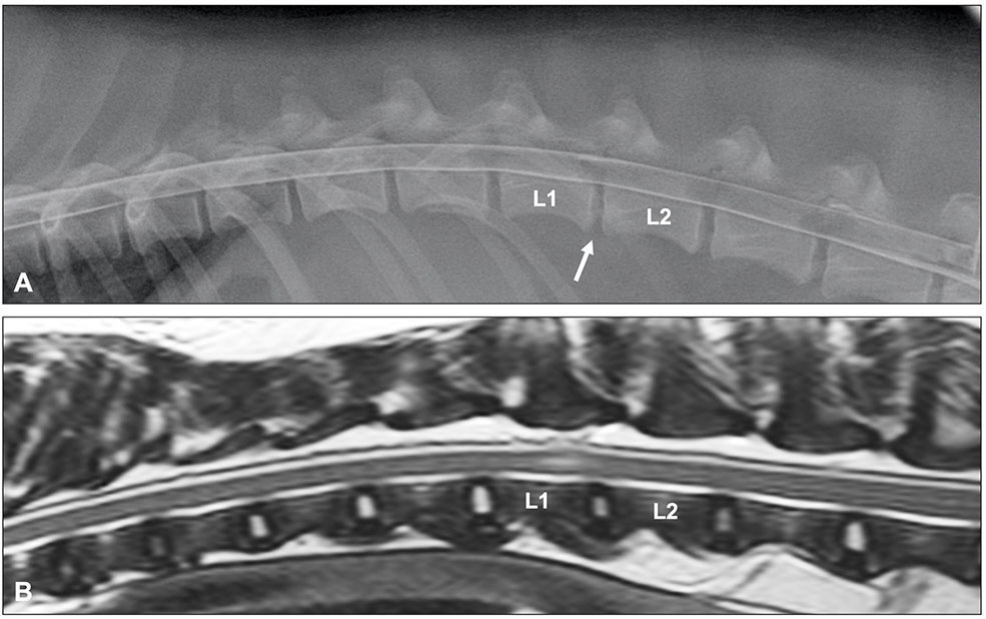
Lateral myelogram **(A)** and sagittal T2-weighted image **(B)** of a 4-year old Soft-Coated Wheaten terrier with an L1-L2 ANNPE. **(A)** Although the L1-L2 intervertebral disc space is narrowed (arrow), the myelographic pattern appears to be normal. **(B)** A focal intramedullary hyperintense lesion just cranial from the L1-L2 disc space can be observed. The homogenously hyperintense nucleus pulposus has a reduced volume and the intervertebral disc space is narrowed.

**Figure 11:**
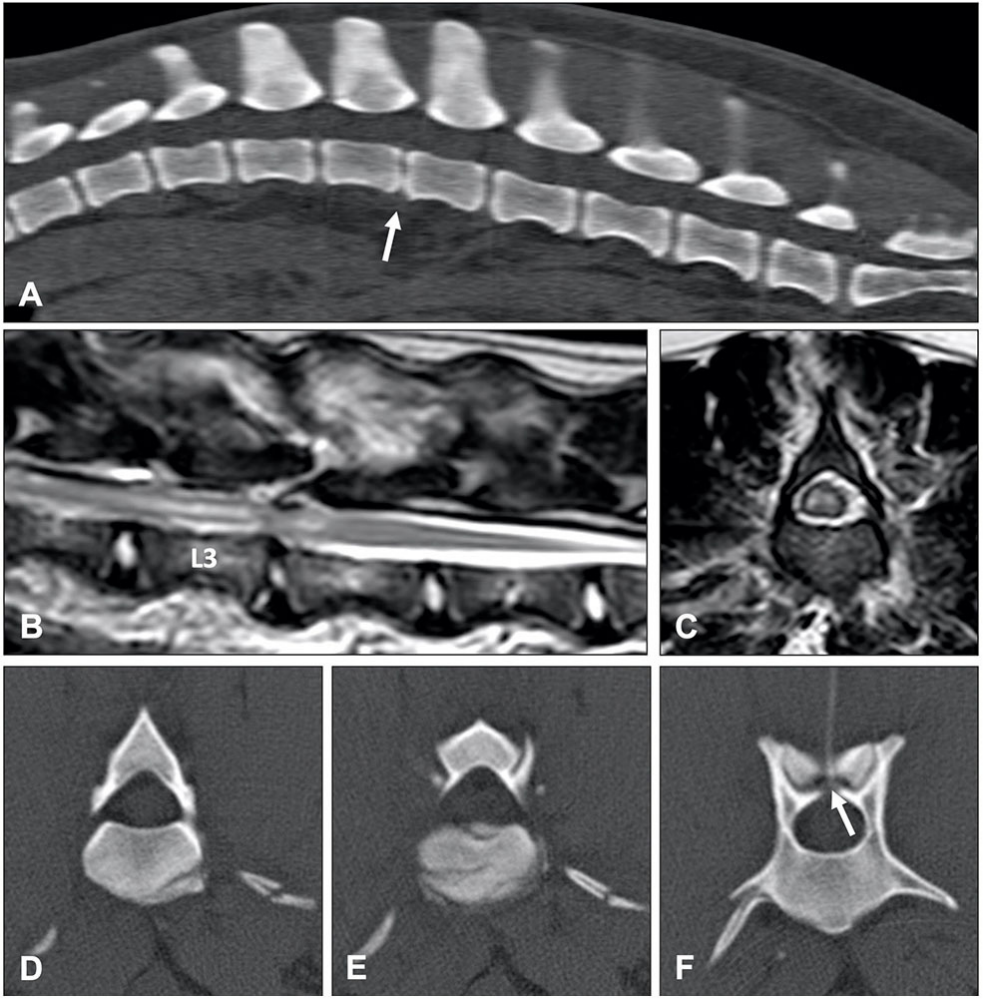
ANNPE can occur after external trauma. CT can be used to exclude or characterize concurrent vertebral fractures and luxations. **(A)** Sagittal reconstructed CT study of a 6-year old Boxer who became paraplegic immediately after a road traffic accident. Although a narrowed intervertebral disc space (arrow) is in itself not sufficient to confirm a diagnosis of ANNPE, the CT study was able to exclude the presence of a vertebral fracture and luxation. Sagittal **(B)** and transverse **(C)** T2-weighted MR images and transverse CT images at the levels of caudal L3 **(D)**, the caudal vertebral endplate of L3 **(E)** and cranial L4 **(F)** of a 4-year old Border collie who ran into a tree. Although the MRI **(A,B)** reveals abnormalities suggestive for ANNPE, there are also abnormalities suggestive for external trauma, such as diffuse and bilateral hyperintensities in the paravertebral muscles. **(C–E)** The CT study revealed multiple fractures and **(D)** a mildly increased distance between the cranial and caudal articular processes.

Survey radiographs can be normal or reveal a narrowed intervertebral disc space in animals with ANNPE. This radiographic finding is not specific and does not help differentiating ANNPE from other types of IVDH. Myelography can reveal a small focal extradural compressive lesion overlying an intervertebral disc space, with an adjacent intramedullary pattern caused by spinal cord swelling (Figure 10) (149). Although it is unclear how specific these findings are for a diagnosis of ANNPE, one study suggested that myelography could reliably be used to differentiate between ANNPE and IVDE (150). An almost perfect interobserver agreement (κ = 0.8) was reached to make a presumptive diagnosis of ANNPE (instead of IVDE) using myelography. The myelographic studies of all dogs with ANNPE demonstrated an intramedullary pattern and an additional extradural pattern was seen in approximately half of dogs. The degree of spinal cord swelling was not associated with severity of clinical signs or outcome (150). Although no specific CT or CT-myelography findings have been reported in dogs with ANNPE, it can be hypothesized that these imaging modalities will show similar abnormalities as survey radiography and myelography, respectively. As mentioned above, ANNPE can be associated with external trauma, such as road traffic accidents or falls from a height. It has been suggested that onset of clinical signs is associated with external trauma in 40% of dogs and more than 70% of cats with ANNPE (147, 151). This observation has two important clinical consequences: (1) ANNPE and vertebral fracture and luxation are two important differential diagnoses in animals suffering spinal cord dysfunction immediately after external trauma, and (2) animals can have concurrent ANNPE and vertebral fracture and luxation after external trauma has occurred (Figure 11). CT is considered the diagnostic modality of choice for animals with suspected vertebral fracture and luxation (152, 153). This diagnostic imaging technique can therefore be utilized in an emergency setting to exclude or characterize a vertebral fracture and luxation after a witnessed or suspected external trauma, but it might miss the presence of ANNPE (Figure 11).

MRI is considered the imaging modality of choice for ANNPE. The following MRI characteristics have been proposed to make a presumptive diagnosis of ANNPE in dogs (147):

- Focal intramedullary, often well-demarcated, spinal cord T2W hyperintensity, which is typically isointense on T1W sequences
- The lesion is located over an intervertebral disc space and is often lateralized
- The nucleus pulposus has a homogenous T2W hyperintense signal and a reduced volume. This is associated with a narrowed intervertebral disc space
- There is a small volume of extradural material dorsal to the affected intervertebral disc space, with minimal to no spinal cord compression. This can be associated with signal changes in the epidural space.

Other less commonly observed MRI changes can include a cleft in the dorsal part of the annulus fibrosus. Mild local enhancement of the meninges or epidural material after IV administration of gadolinium-based contrast has been reported (154), though this is usually not present (147, 150). Additionally, although more commonly observed in dogs with IVDE, signal alterations in the paravertebral muscles can also occasionally be seen in dogs with ANNPE (112). Paravertebral signal changes are occasionally seen with ANNPE and appear similar to what is described for IVDE (112).

**Figure 12:**
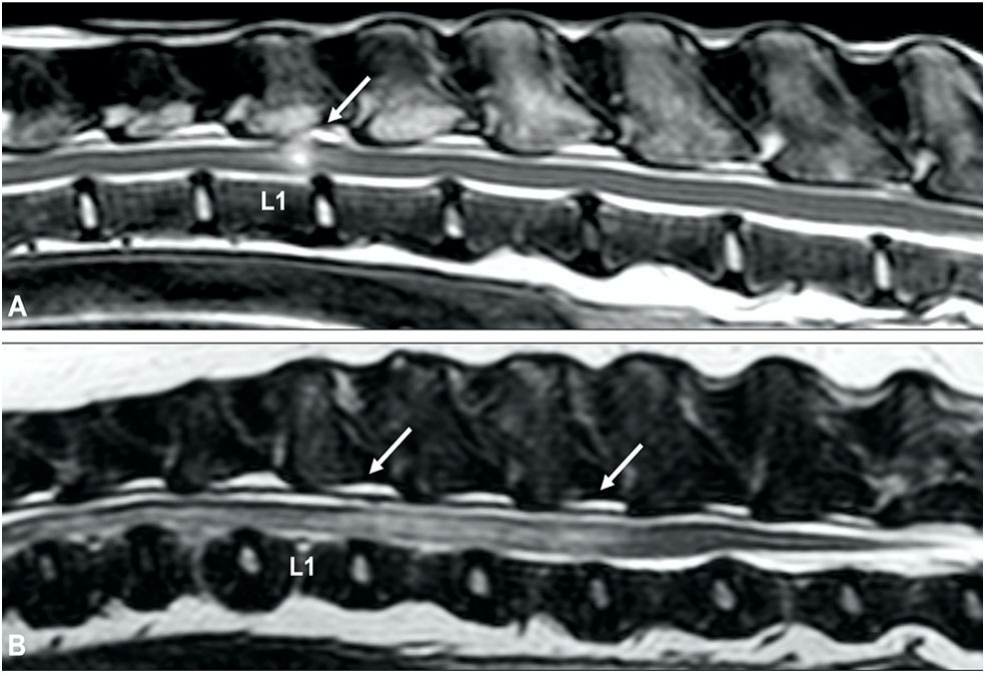
T2-weighted sagittal MR images of **(A)** an 8-year old cross breed with ANNPE and **(B)** a 4-year old Staffordshire Bull terrier with ischemic myelopathy. **(A)** Focal hyperintensity can be seen overlying the L1-L2 intervertebral disc. The L1-L2 intervertebral disc space is narrowed and has a reduced volume of T2W hyperintense nucleus pulposus. **(B)** A relatively well-demarcated longitudinal hyperintensity can be seen between L1 and L4.

The clinical presentation of animals with ANNPE is almost identical to that of animals with ischemic myelopathy, which is most commonly caused by fibrocartilaginous embolic myelopathy. Differentiating between both conditions is important because there are indications that the long-term outcome, and especially the prevalence of fecal incontinence, might be different between dogs with ANNPE and ischemic myelopathy (155, 156). Similar to ANNPE, specific MRI characteristics have been reported for ischemic myelopathy, which include a focal, relatively well-demarcated, possibly lateralized, longitudinal T2W hyperintense intramedullary lesion primary affecting the gray matter. The length of the lesion is usually longer than one vertebral body length (Figure 12) (157). Although it is possible to presumptively differentiate these two conditions using MRI, there is considerable overlap between MRI appearance of dogs with ANNPE and ischemic myelopathy. Studies evaluating the interobserver agreement to differentiate ANNPE and ischemic myelopathy based on MRI characteristics have produced variable results reporting moderate (κ = 0.56) to perfect (κ = 1) interobserver agreement (158, 159). A lesion overlying the intervertebral disc, lesion lateralization, reduced nucleus pulposus volume, presence of extradural signal changes, meningeal enhancement and a non-longitudinal directional pattern of hyperintensity on T2W images have been associated with a diagnosis of ANNPE instead of ischemic myelopathy. A lesion overlying the vertebral body and greater length of an intramedullary hyperintensity were associated with a diagnosis of ischemic myelopathy instead of ANNPE (155, 159). A limitation to the literature on the subject is the lack of histopathologic confirmation of imaging findings.

Abnormalities observed on MRI in dogs with ANNPE have also been associated with the functional outcome (147, 160). Larger lesions observed on transverse sections have been associated with an unsuccessful outcome (147, 160). More information regarding MRI and outcome is presented in the manuscript “Prognostic factors in acute spinal cord injury” in this issue.

## Hydrated Nucleus Pulposus Extrusion

Hydrated nucleus pulposus extrusion (HNPE) is characterized by sudden extrusion of minimally to non-degenerative nucleus pulposus. Well-hydrated, gelatinous, extradural material can be identified in the vertebral canal, which is associated with varying degrees of spinal cord compression (148). Although HNPE can occur in the thoracolumbar region, it has a predilection for the cervical vertebral column (145). The most common clinical presentation consists therefore of acute onset non-ambulatory tetraparesis. Cervical hyperesthesia is not commonly observed. In contrast to dogs with ANNPE, onset of clinical signs is typically not associated with vigorous exercise or external trauma (161).

**Figure 13:**
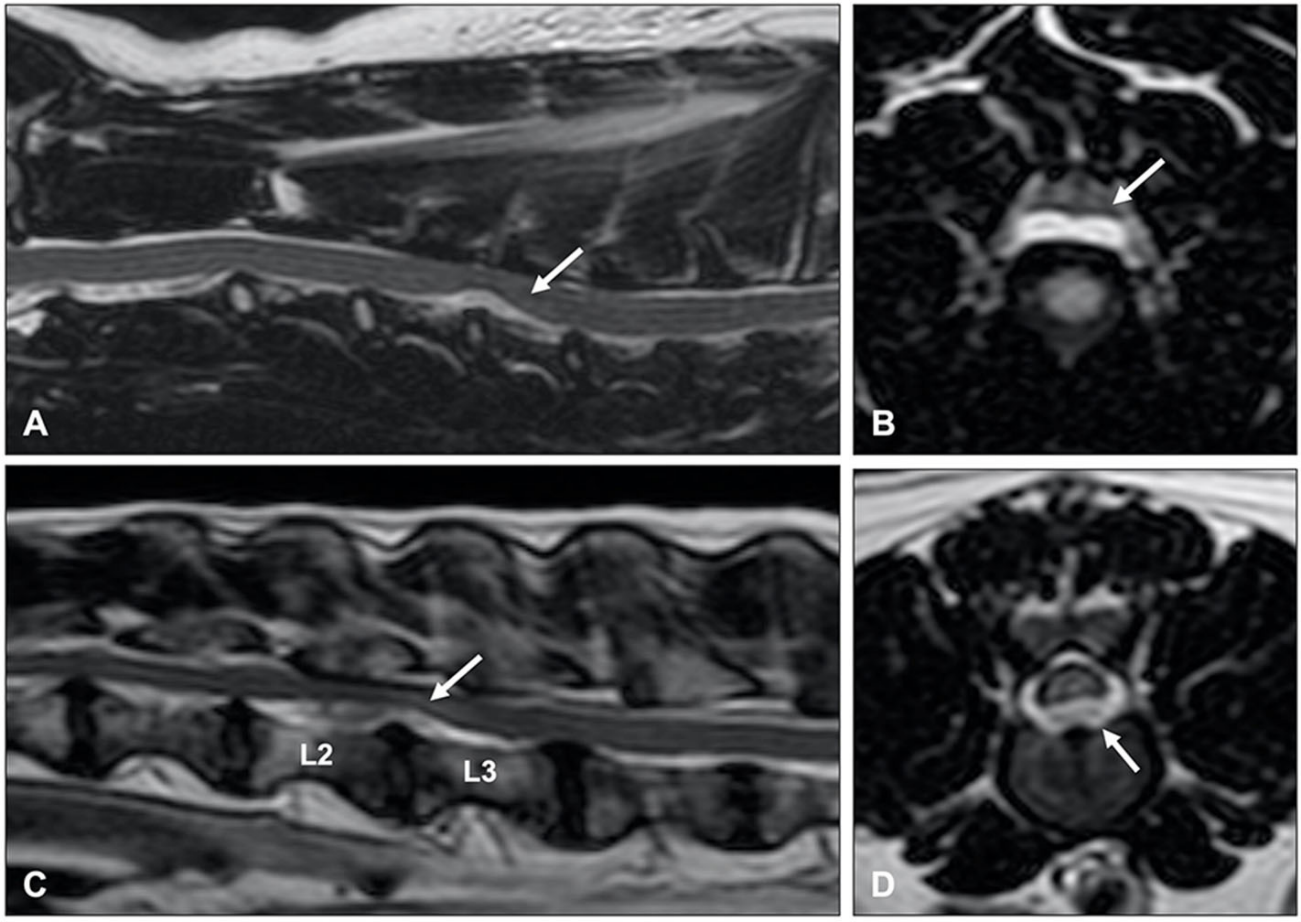
T2-weighted **(A)** sagittal and **(B)** transverse MR images of a 6-year old Border collie with a C4-C5 HNPE. The intervertebral disc space is narrowed and contains a reduced volume of hyperintense nucleus pulposus. There is a ventral extradural homogenously hyperintense compressive lesion. **(B)** The compressive material is midline and has a bilobed, “seagull” appearance. T2-weighted **(C)** sagittal and **(D)** transverse images of 7-year old Beagle with acute onset paraplegia. Although HNPE occurs most often in the cervical region, this dog was diagnosed with an L2-L3 HNPE.

Magnetic resonance imaging is the diagnostic modality of choice and specific, almost pathognomonic, MRI abnormalities have been described (Figure 13) (148). The typical MRI appearance of HNPE is characterized by:

- A narrowed intervertebral disc space with a reduced volume of hyperintense nucleus pulposus (148)
- Ventral, midline, extradural compressive material, which is homogenously hyperintense on T2W sequences and isointense in all sequences to normal, non-degenerative nucleus pulposus lying immediately dorsal to the affected intervertebral disc
- The extruded material can have a characteristic bilobed or “seagull appearance.” This typical shape is possibly explained by the location of the compressive material just ventral to the intact dorsal longitudinal ligament (162).
- A T2W hyperintense intraparenchymal lesion can be present in the overlying spinal cord and extruded nucleus pulposus can demonstrate a degree of contrast enhancement (148, 162).

Similar to ANNPE, MRI findings have been linked to the likelihood of neurological recovery in dogs with HNPE. Although T2W hyperintense intraparenchymal lesions are present in less than half of dogs with HNPE (163, 164), the length of such lesions was negatively associated with the likelihood for functional recovery within a time period of 9 days (164).

Although MRI is preferred, one study evaluated the usefulness of CT to diagnose cervical HNPE (165). Non-contrast CT did not reveal any specific abnormalities, however CT-angiography did allow visualization of cervical HNPE as a well-demarcated hypodense extradural compressive lesion with rim enhancement immediately dorsal to the intervertebral disc space. Contrast enhanced CT had a sensitivity of 91% and specificity of 100% to differentiate between cervical HNPE and IVDE (165).

## Intradural/Intramedullary Intervertebral Disc Extrusion

**Figure 14:**
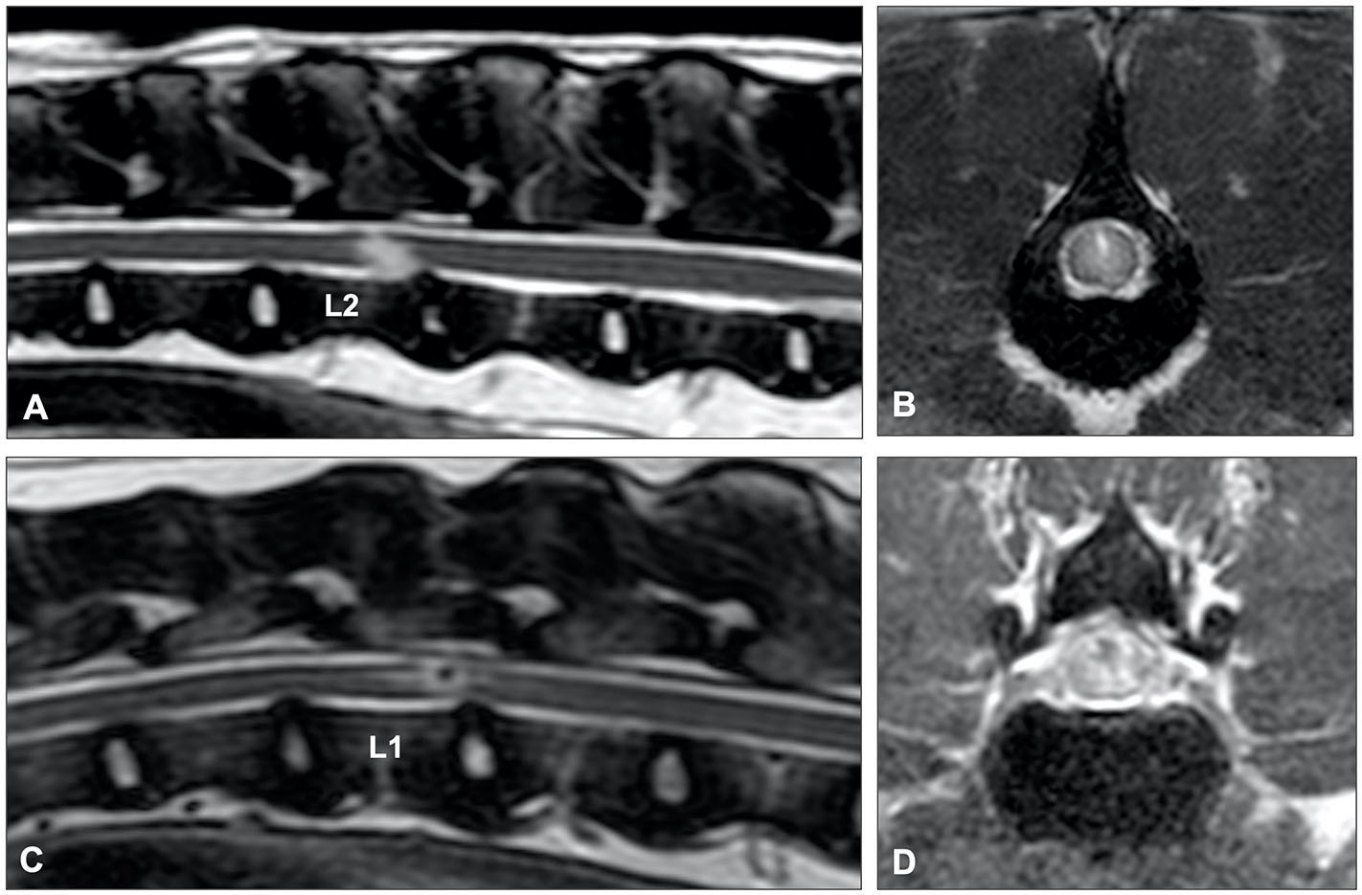
**(A)** T2-weighted sagittal and **(B)** BALT GRAD (T2-weighted thin-slice gradient echo) transverse MR images of an 8-year old Greyhound with an L2-L3 IIVDE. **(A)** The intervertebral disc has a decreased volume of homogenously hyperintense nucleus pulposus. A ventrocaudal to dorsocranial intramedullary linear hyperintensity, starting from the L2-L3 intervertebral disc can be seen. **(B)** Hyperintense linear tract can be seen through the spinal cord. **(C)** T2-weighted sagittal and **(D)** BALT GRAD transverse MR images of a 6-year old Beagle with an L1-L2 IIVDE. The intramedullary lesion has a hypointense center, suggestive for intraparenchymal hemorrhage.

Intradural/intramedullary intervertebral disk extrusion (IIVDE) is the least common type of herniation of nucleus pulposus. This type of disc extrusion is characterized by intradural or intramedullary extrusion of calcified and dehydrated or minimally degenerate and hydrated intervertebral disc material (6, 146). The variation in hydration status of extruded material in dogs with IIVDE can result in variable clinical signs and imaging findings. Affected dogs can have a similar clinical presentation as dogs with ANNPE and ischemic myelopathy (146) or can present with a similar presentation as dogs with IVDE (6). Similar to ANNPE, MRI in animals with IIVDE caused by minimally degenerate nucleus pulposus can demonstrate a narrowed intervertebral disc space, decreased volume of homogenously hyperintense nucleus pulposus, and a T2W hyperintensity dorsal to the affected intervertebral disc space. Specific MRI findings in animals with IIVDE include a linear tract (predominantly hyperintense on T2W images, iso to hypointense on T1W images and hypointense on T2^*^-weighted GRE images) extending from the intervertebral disc into the spinal cord parenchyma (Figure 14) (146). Mild enhancement of the tissue adjacent to the tract can be observed on T1W images after IV administration of gadolinium-based contrast medium. Similar to IVDE, MRI in dogs with IIVDE caused by dehydrated and calcified nucleus pulposus can demonstrate a narrowed and homogenously hypointense intervertebral disc space with a hypointense mass dorsal to the affected intervertebral disc space, resulting in obvious spinal cord compression. Hyperintense lesions on T2W images, possibly indicating leakage of CSF, can be observed surrounding the herniated material. CT-myelography has been demonstrated to be particularly useful for diagnosing IIVDE and has been suggested to be more sensitive than low-field MRI for this purpose (6). CT-myelography can reveal extruded intervertebral disc material within the subarachnoid space with focal accumulation of contrast media within the subarachnoid space. The filling defect associated with the intradural location of the lesion can appear relative small in size predicted by the accumulation of contrast medium (6). It has been suggested that visualization of extradural leakage, suggestive of a dural tear, can be improved by applying traction to the head (166).

## Novel Advanced Imaging Techniques in IVDD

### Diffusion Tensor Imaging and Tractography

While conventional advanced imaging (MRI, CT, less commonly, myelography) are indispensable in the diagnosis of canine IVDH, there is a growing role for specialized applications of MRI including diffusion tensor imaging (DTI) in evaluating this population.

**Figure 15:**
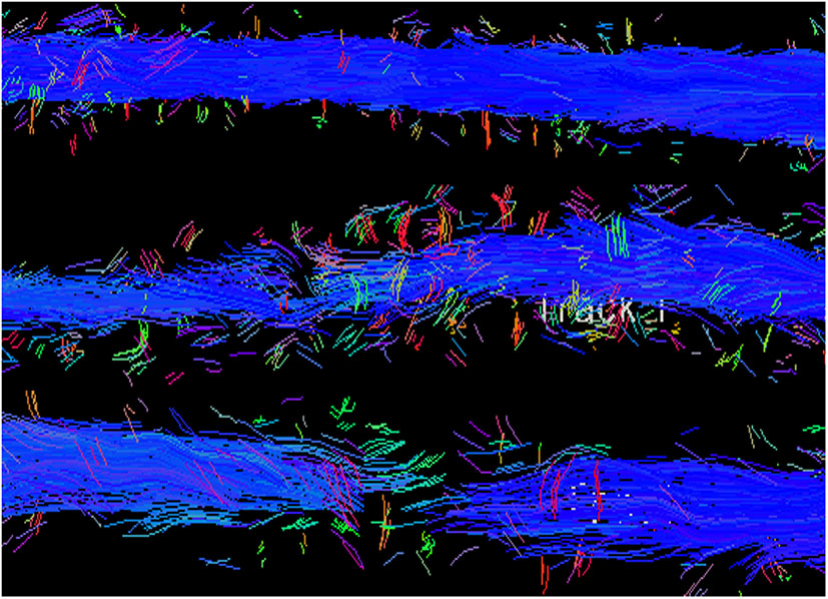
Tractography of the spinal cord from a normal dog (top) compared to dogs with SCI showing moderate fiber disruption (middle) and complete trans-lesional discontinuity (bottom). Blue depicts cranial to caudal oriented fibers.

Diffusion tensor imaging is a variation on diffusion weighted imaging that relies on the strength and direction of cellular diffusion of water molecules to create images (167–169). The movement of water molecules varies by tissue type and is altered by pathology allowing DTI to provide quantitative microstructural information. Quantitative analysis typically includes calculation of the fractional anisotropy (FA) and apparent diffusion coefficient (ADC) or mean diffusivity (MD). Fractional anisotropy approximates the degree of directional dependence within a region from isotropic (0) to completely anisotropic along a single axis, providing information on white matter integrity. Apparent diffusion coefficient or MD relate to the magnitude of diffusion within a given region measured as the rate of water motion and reflect global tissue architecture. Axial diffusivity (AD) and radial diffusivity (RD) are performed less commonly and refer to diffusivity in the direction of or perpendicular to fiber tracts, respectively. Axial diffusivity and RD have been inconsistently reported to be associated with axonal injury and demyelination, respectively (169–172). The quantitative information on diffusivity within each voxel can then be combined to create a visual representation of white matter tracts called tractography (Figure 15) (173, 174).

Spinal cord DTI is useful to quantify disruption of the normally highly anisotropic white matter tracts and can detect microstructural changes such as axonal damage, demyelination, Wallerian degeneration and loss of tissue architecture (169). In experimental rodent models and human SCI patients, DTI has been shown to be more sensitive to pathologic changes than conventional MRI sequences (174–179). Additionally, quantitative indices and tractography have also been variably associated with injury severity, spinal cord integrity and long-term functional outcome in people and rodent models of SCI (176–185).

### Diffusion Tensor Imaging of the Spinal Cord in Dogs

The feasibility of spinal cord DTI has been established in neurologically normal dogs (186–189). Healthy Beagles and Dachshunds predominated (186, 188, 189), but Hobert et al. included dogs of various ages, breeds and body sizes (187). No association was identified between FA or ADC values and body size or the cranial to caudal location along the spinal cord; age was not specifically investigated (187). These studies provide useful protocol and scan time information and broad reference values for quantitative variables in the uninjured cervical and thoracolumbar spinal cord.

Diffusion tensor imaging has been reported in dogs with experimental injury and various naturally-occurring myelopathies (118, 188, 190–196). Among experimental models, DTI indices have been correlated with specific histologic changes after injury, supporting its potential utility as a non-invasive measure of spinal cord microstructure (193, 195, 196). In dogs with naturally-occurring SCI, the majority of which were due to IVDE, quantitative analysis and tractography were able to distinguish between normal controls and dogs with SCI and between acute and chronic injury (118, 188, 194). In general, FA is reported to be decreased within and adjacent to an area of injury compared to controls and is further decreased in chronic relative to acute injury (118, 188, 191, 192). Values of ADC/MD within and adjacent to the lesion are more variable, likely influenced by the variable imaging timing across studies (118, 188, 191, 192, 194). Additionally, ADC/MD values can be decreased in acute SCI but become significantly elevated (relative to acute injury or healthy control dogs) in the chronic setting (118, 191, 192). Consistent with studies in people and experimental models, DTI in dogs has also been able to detect abnormal areas that appear macroscopically normal on T2W sequences, improving delineation of the regional extent of the SCI beyond what is visible with standard MRI (192).

Diffusion tensor imaging has been explored in dogs as a potential non-invasive biomarker and prognostic indicator (118, 192, 197). In dogs with paraplegia secondary to IVDE who underwent decompressive surgery, FA was not shown to be a better predictor of functional outcome compared to initial assessment of pain perception status (118). However, for dogs imaged in the chronic timeframe after prior acute injury, higher FA was associated with greater pelvic limb motor function, suggesting an association between diffusivity, structure and function (192). Additionally, among paraplegic dogs with or without pain perception secondary to IVDE, MD might be useful to detect acute injury severity and to predict outcome (197). Tractography has also been shown to detect loss of spinal cord integrity including two dogs with IVDE (Figure 15) (192). Based on the small number of current studies, the utility of DTI and tractography to quantify injury severity and predict prognosis remains unclear. Further evaluation focusing on prospective studies in a larger number of dogs with IVDH is warranted.

In spite of the potential advantages, DTI faces several technical and logistical considerations that generally impede widespread use in dogs with SCI. DTI also requires specialized software, technical expertise and extensive post-processing further limiting its utility in real-time, clinical decision making for dogs with acute IVDH. Standardizing and optimizing acquisition and processing protocols will be important in order for DTI to be incorporated into routine clinical use in SCI patients. Despite limitations, DTI is worthy of continued development to complement standard MRI studies in cases of acute and chronic IVDH in dogs.

### Additional MRI-Based Spinal Cord Imaging Techniques

Other novel advanced imaging techniques have been evaluated in the spinal cord of people including magnetic resonance spectroscopy, magnetization transfer imaging, myelin water fraction imaging and functional MRI (178, 198–201). These imaging modalities have been reported for the canine brain, in dogs with cervical spondylomyelopathy and in an experimental canine disc degeneration model but have not yet been investigated in dogs with IVDH (104, 202, 203). Future studies in canine IVDH should be considered to assess their clinical and translational utility in this population.

## Conclusion

This review outlined the available imaging modalities used in the diagnosis of all forms of IVDD including IVDE, IVDP, ANNPE, HNPE, and IIVDE. Radiographs remain a screening tool but have limitations, especially with regard to differentiating between IVDD subtypes and offering a definitive diagnosis. Myelography, CT, or MRI are all viable ways to diagnose IVDD, with CT and MRI largely having supplanted myelography in routine clinical practice. While there are pros and cons for both CT and MRI, patient selection is the most important factor when choosing the appropriate imaging modality to maximize the likelihood of achieving a definitive diagnosis. Non-contrast CT is a quick and economical choice that is highly likely to be successful in an acutely non-ambulatory, chondrodystrophic dog where there is high suspicion for IVDE, with CT-myelography required in selected cases. Magnetic resonance imaging is considered the gold standard imaging modality for acute and chronic spinal cases due to its ability to allow spinal cord visualization and the diagnosis of IVDE, IVDP, ANNPE, HNPE, or IIVDE. There are a growing number of studies of DTI in dogs with IVDD that offer a means to assess microstructural lesion characteristics and spinal cord integrity. It is likely that additional, novel spinal cord imaging techniques will be developed for application in dogs with IVDD. Overall, diagnostic imaging is indispensable in the diagnosis of IVDD and a keen knowledge of the advantages and limitations of the various imaging modalities is crucial to maximize the diagnostic information obtained.

## Author Contributions

RC, SD, ML, and HV contributed to manuscript concept, preparation, and editing. The additional members of the CANSORT-SCI contributed to manuscript concept, editing, and review. All authors contributed to the article and approved the submitted version.

## Conflict of Interest

The authors declare that the research was conducted in the absence of any commercial or financial relationships that could be construed as a potential conflict of interest.
